# RUNX1 Regulates a Transcription Program That Affects the Dynamics of Cell Cycle Entry of Naive Resting B Cells

**DOI:** 10.4049/jimmunol.2001367

**Published:** 2021-12-15

**Authors:** Inesa Thomsen, Natalia Kunowska, Roshni de Souza, Anne-Marie Moody, Greg Crawford, Yi-Fang Wang, Sanjay Khadayate, Chad Whilding, Jessica Strid, Mohammad M. Karimi, Alexis R. Barr, Niall Dillon, Pierangela Sabbattini

**Affiliations:** * Gene Regulation and Chromatin Group, MRC London Institute of Medical Sciences, London, United Kingdom;; † Department of Immunology and Inflammation, Imperial College London, London, United Kingdom;; ‡ Bioinformatics and Computing, MRC London Institute of Medical Sciences, London, United Kingdom;; § Microscopy Facility, MRC London Institute of Medical Sciences, London, United Kingdom;; ¶ Comprehensive Cancer Centre, School of Cancer & Pharmaceutical Sciences, King’s College London, London, United Kingdom;; ‖ Cell Cycle Control Group, MRC London Institute of Medical Sciences, London, United Kingdom; and; # Institute of Clinical Sciences, Imperial College London, London, United Kingdom

## Abstract

RUNX1 is a transcription factor that plays key roles in hematopoietic development and in hematopoiesis and lymphopoiesis. In this article, we report that RUNX1 regulates a gene expression program in naive mouse B cells that affects the dynamics of cell cycle entry in response to stimulation of the BCR. Conditional knockout of *Runx1* in mouse resting B cells resulted in accelerated entry into S-phase after BCR engagement. Our results indicate that Runx1 regulates the cyclin D2 (*Ccnd2*) gene, the immediate early genes *Fosl2*, *Atf3*, and *Egr2*, and the Notch pathway gene *Rbpj* in mouse B cells, reducing the rate at which transcription of these genes increases after BCR stimulation. RUNX1 interacts with the chromatin remodeler SNF-2–related CREB-binding protein activator protein (SRCAP), recruiting it to promoter and enhancer regions of the *Ccnd2* gene. BCR-mediated activation triggers switching between binding of RUNX1 and its paralog RUNX3 and between SRCAP and the switch/SNF remodeling complex member BRG1. Binding of BRG1 is increased at the *Ccnd2* and *Rbpj* promoters in the *Runx1* knockout cells after BCR stimulation. We also find that RUNX1 exerts positive or negative effects on a number of genes that affect the activation response of mouse resting B cells. These include *Cd22* and *Bank1*, which act as negative regulators of the BCR, and the IFN receptor subunit gene *Ifnar1*. The hyperresponsiveness of the *Runx1* knockout B cells to BCR stimulation and its role in regulating genes that are associated with immune regulation suggest that RUNX1 could be involved in regulating B cell tolerance.

## Key Points

- RUNX1 reduces cell cycle and immediate early gene expression in naive B cells.
- BCR stimulation triggers a switch from RUNX1 to RUNX3 binding at cell cycle genes.
- Notch signaling is downregulated by RUNX1 in follicular B cells.

## Introduction

The pioneer transcription factor, RUNX1, is involved in the initiation of hematopoiesis at a very early stage in mammalian development and is required for fetal liver hematopoiesis and maintenance of the correct balance of adult hematopoietic stem cell compartment formation (1–4). In addition to its roles in hematopoiesis, RUNX1 also has critical roles in the early stages of T and B cell development (3, 5). RUNX1 belongs to the runt-related family of transcription factors, which has three members, RUNX1, RUNX2, and RUNX3. The runt-related factors act in conjunction with the non-DNA binding factor, CBFβ, to regulate gene expression in development, cell differentiation, and cancer (reviewed in Ref. 2).

The biological effects of RUNX1 are highly context dependent, and it has been shown to function either as a transcriptional activator or as a repressor through interactions with different cofactors (6). This diversity of function is also reflected in the fact that RUNX1 can act as a tumor suppressor or as an oncogene in different cell types (7). The *RUNX1* gene is involved in multiple translocations that give rise to oncogenic fusion proteins (8). These include the *RUNX1*-ETO fusion, which is the most common cytogenetic abnormality in acute myeloid leukemia (9), and the ETV6-*RUNX1* fusion, which occurs in ∼25% of cases of childhood precursor B cell acute lymphoblastic leukemia (10, 11).

During early B cell development, RUNX1 acts in conjunction with E2A and early B cell factor (EBF) to activate B cell–specific gene expression at the pre-pro-B cell stage (12, 13), and conditional knockout (c-k/o) of *Runx1* in pro-B cells results in a block in the transition from the pro- to the pre-B cell stage (13). In human mature B cells, analysis of the functions of RUNX1 and RUNX3 in EBV-transformed B cells has shown that the EBV transcription factor, EBNA2, enhances expression of RUNX3, resulting in RUNX3-mediated downregulation of *RUNX1* expression (14). Human RUNX1 was also shown to have a growth-inhibitory effect on EBV-transformed cells, which was not observed for mouse RUNX1 because of the absence of a specific N-terminal segment from the mouse protein (15). Despite these results indicating a role for RUNX1 in regulating B cell proliferation, there is very little information about the mechanisms by which this might occur or how RUNX1 affects naive B cell activation in response to antigenic stimulation of the BCR.

In this article, we show that RUNX1 acts in conjunction with the chromatin remodeling complex SNF-2–related CREB-binding protein activator protein (SRCAP) to regulate a gene expression program that affects the timing of entry of mouse resting B cells into S-phase. RUNX1 binds to promoters and distally located elements at key cell cycle and immediate early genes that have a poised epigenetic configuration. c-k/o of the *Runx1* gene in resting B cells results in deregulation of Cyclin D2 and immediate early gene expression, enhanced Notch signaling, and accelerated entry into S-phase in response to BCR stimulation. We also show that RUNX1 is responsible for correct regulation of genes that affect B cell survival, functioning of the BCR, and the response to IFN.

## Materials and Methods

### Mice

The Runx1 c-k/o/Cd23-Cre mice were generated by crossing the Runx1^fl/fl^ mouse line (generously provided by Nancy Speck, University of Pennsylvania) (16) with the Cd23-Cre transgenic mouse line (generously provided by Meinrad Busslinger, IMP Vienna) (17). The CD23-cre transgene is first expressed at the transitional (T) stage, resulting in c-k/o of floxed target genes in mature resting B cells (17). All work involving mice was conducted under the regulations of the British Home Office and was approved by the Imperial College Animal Welfare and Ethical Review Body.

### Spleen and lymph node isolation and splenic resting B cell purification and activation

Spleens and cervical lymph nodes were isolated from 6- to 10-wk-old C57B6 mice and homogenized through a sieve in B cell culture medium (RPMI-1640 [Lonza], 10% FCS [Sigma], 0.1 U/ml penicillin [Lonza], 0.1 μg/ml streptomycin [Lonza], 2 mM l-glutamine [Lonza], and 50 μM 2-ME [Life Technologies]). For large-scale preparations for chromatin immunoprecipitation (ChIP) sequencing (ChIP-seq), the cell suspension was centrifuged on a Ficoll-Paque (GE Healthcare) cushion, and the buffy coat layer was resuspended at a concentration of 1× 10^8^ cells/ml in PBS + 2% FCS/1 mM EDTA. Resting B cells were isolated using the EasySep Negative Selection Mouse B Cell Isolation Kit (STEMCELL Technologies), which depletes for non-B cell markers and activated CD43^+^ B cells. For activation, purified resting B cells were cultured at a density of 1.5–2 × 10^6^/ml in B cell culture medium supplemented with 25 mg anti-IgM Ab (Millipore) and 2 ng/ml IL-4 (PeproTech) for 20 h. LPS (Sigma) was then added to a final concentration of 25 μg/ml followed by incubation for 1–6 h. A concentration of 25 μg/ml LPS for 34 h was used for LPS-only activation. Incubation with anti-IgM (25 μg/ml) + IL-4 (2 ng/ml) + anti-CD40 (20 μg/ml; ENZO) for 26 h was used to provide costimulatory activating conditions. For cell viability assays in the presence of BAFF, purified resting B cells were incubated in culture medium with soluble BAFF (1 mg/ml; ENZO) for 72 h. For γ-secretase inhibition experiments, resting B cells were precultured in B cell culture medium with 20 μM DAPT (Sigma) or with vehicle (DMSO) for 4 h followed by addition of anti-IgM + IL-4.

### FACS analysis

Cells (1× 10^6^) were collected by centrifugation and washed twice in PBS/2% FCS, and the cell pellet was resuspended in 100 μl of PBS/2% FCS. Fluorophore-conjugated surface marker Abs were added at a 1:100 dilution and incubated on ice for 20 min. Cells were washed in PBS/2% FCS and resuspended in PBS/2% FCS for analysis on a BD LSR II Flow Cytometer. The Abs used in the FACS analysis were as follows: CD21-FITC (553818) BD Biosciences Rat, CD23-PE (561773) BD Pharmingen Rat, CD23-Pacific blue (101616) BioLegend Rat, IgD-allophycocyanin (405713) BioLegend Rat, IgM-PE-Cy7 (406513) BioLegend Rat, B220-Pacific blue (558108) BD Biosciences Rat, B220-PE (553089) BD Biosciences Rat, and CD22-PE (BD 553384). The LIVE/DEAD Fixable Aqua Dead Cell Stain Kit (Thermo Fisher Scientific) was used to distinguish between live and dead cells. Data were analyzed with FlowJo software. The gating strategy was that cells were initially gated for live lymphocytes followed by analysis of B220^+^ cells.

### FITC Annexin V/Dead Cell Apoptosis Kit

Cells (1 × 10^6^) were collected by centrifugation and washed in ice-cold PBS. A FITC Annexin V/Dead Cell Apoptosis Kit (Invitrogen), containing FITC-conjugated Annexin V and propidium iodide (PI), was used to analyze the rate of apoptosis and death in cells. Cells were analyzed on a BD LSR II Flow Cytometer.

### Cell cycle analysis by PI staining

Cells (1 × 10^6^) were collected by centrifugation and washed with PBS/2% FCS. The cell pellet was resuspended in 50 μl of PBS/2% FCS. A volume of 500 μl of ice-cold 70% ethanol was added to the cell suspension, followed by 10-min incubation on ice. The sample was centrifuged and washed twice in PBS/2% FCS. The pellet was resuspended in 50 μl of PBS/2% FCS and incubated for 30 min in the dark in 500 μl of PI solution (PBS, 0.05 mg/ml PI [Sigma], 0.05% Nonidet P-40 [Sigma], and 1 μg/ml RNase A [Sigma]). Samples were analyzed on a BD LSR II Flow Cytometer (BD Biosciences).

### RNA analysis

B cells (3 × 10^6^) were pelleted by centrifugation and resuspended in 0.3 ml of TRIzol (Thermo Fisher Scientific), and RNA spike-in (generated by in vitro transcription; sequence is available on request) was added at a concentration of 0.1 ng/1 × 10^6^ cells. After RNA purification using the RNeasy mini kit (Qiagen), reverse transcription of 200 ng of RNA was carried out using the SuperScript II reverse transcriptase kit (Thermo Fisher). Real-time quantitative PCR (RT-qPCR) analysis was carried out using the SensiMix SYBR No-Rox kit (Bioline). Primer sequences and PCR conditions are available on request. RNA levels were normalized using the following equation: (Gene 2^−Ct^/spike 2^−Ct^) = gene expression relative to spike.

### RNA sequencing

B cells (30 × 10^6^) were lysed in 1 ml of TRIzol, and 30 μl of 1:100 diluted ERCC RNA Spike-In Mix (Ambion) was added to each lysate. RNA was isolated and eluted in a final volume of 20 μl. One microliter of each sample was used for quality and concentration analysis on a 2100 Bioanalyzer using the RNA 6000 Nano kit (Agilent). A total of 500 ng of RNA was used to prepare each mRNA library. PolyA RNA selected RNA Libraries were generated by the MRC London Institute of Medical Sciences Core Genomics Facility using the TruSeq Stranded mRNA Library Prep Kit (Illumina). Paired-ended sequencing was performed on an Illumina HiSeq 2500 sequencer. The reads were aligned to mouse genome mm9 using Tophat2 with default parameters and gene annotation from Ensembl version66. Genome-wide coverage of the RNA sequencing (RNA-seq) datasets was generated as bedGraph files using BEDTools and converted to Bigwig files. Read counts on genes were computed using featureCounts function in Rsubread R package. Differentially expressed genes (DEGs) were identified using DESeq2 considering the covariates of interest and factors of unwanted variation computed using RUV-seq.

### Gene set enrichment analysis

Mouse gene symbols were converted to human gene symbol using Ensembl Biomart gene set enrichment analysis (GSEA 2.2.0) was then performed with GseaPreranked tool using Hallmark and C2 Canonical Pathway gene sets.

### Quantitative single-cell imaging

Cells (60,000/well in 20 μl) were deposited in 384-well plates (CellCarrier 384-ultra; Perkin Elmer) coated with 0.01% polylysine and processed as follows: spin 800 × *g* for 1 min; fixation in 2% paraformaldehyde/PBS for 15 min at room temperature; 3× 3-min wash with PBS; permeabilization with 0.3% Triton X/PBS for 15 min; 3× 3-min wash with PBS; blocked overnight with 0.2% fish gelatin (Sigma)/5% horse serum/PBS (block solution); first Ab staining in block for 1 h at room temperature. Then, 2× 3-min wash with PBS/0.2% fish gelatin and 1× 3-min wash with PBS; second Ab staining in block for 1 h at room temperature. Finally, 1× 3-min wash with PBS/0.2% fish gelatin and 1× 3-min wash with PBS; Hoechst staining for 15 min at room temperature (in PBS, 5 μg/ml final); 2× 3-min wash with PBS. Cells were kept in PBS/0.05 Na azide and sealed with foil at 4°C. Fixed and immunostained cells were imaged on the Operetta HCS CLS (PerkinElmer) with a 40× water immersion objective, NA 1.1. Quantitative, automated image analysis was performed using Harmony software (PerkinElmer). Nuclei were detected and segmented based on Hoechst intensity, and nuclei touching the edge of the field were filtered out. Clumps of nuclei and dead nuclei were excluded based on nuclear area and Hoechst intensity such that only single nuclei were included in the analyses. The intensity of nuclear proteins was quantified using the nuclear segmentation mask. Due to B cells having a very small cytoplasm, intensities of cytoplasmic proteins were quantified by segmenting a ring around the edge of the nuclear segmentation mask and three pixels wide (referred to as the “ring region”). Single-cell and average well data were plotted using Prism 8 software (GraphPad).

### ChIP and ChIP-seq

ChIP analysis of resting B cell was performed on formaldehyde-fixed cells as for ChIP-seq described by Sabbattini et al. (18), with the following Abs: RUNX1 (ab23980; Abcam), SRCAP (NBP145244; Novus Biologicals), H3K27me3 (9733S; NEB), RING1B (D139-3; MBL), RUNX3 (ab135248; Abcam), BRG1 (ab110641; Abcam), H2A.Z (ab150402; Abcam), Normal Rabbit IgG (2729S; Cell Signaling Technologies), and H3K4me3 (07-473; Merck Millipore). Quantitative PCR (qPCR)-ChIP was carried out using the SensiMix SYBR No-Rox kit (Bioline). Primers and conditions are available on request. For ChIP-seq, 5 ng of ChIP/Input DNA was used to prepare libraries using the NEBNextUltra II DNA Library Prep Kit following the manufacturer’s recommendations. Libraries were sequenced on an Illumina Hiseq2500 (v4 chemistry). ChIP-seq tracks represent pooled sequences from three independent replicates, with the exception of the SRCAP track, which was generated from two independent replicates. Paired-end reads were aligned to University of California Santa Cruz (UCSC) mm9 mouse genome (https://emea.support.illumina.com/sequencing/sequencing_software/igenome.html) using bowtie2 (2.3.4). Single-end reads were aligned to mm9 mouse genome with BWA (0.7.5a). Aligned reads were sorted and converted to bam files by samtools (1.6). Duplicated reads were removed by Picard MarkDuplicates tool (1.9; Picard Toolkit, 2019) after merging the biological replicates with samtools. Read profiles were normalized to reads per million with bedtools, and bedGraphToBigWig obtained from UCSC. The UCSC reads per million tracks were generated for visualization on UCSC genome browser. Peak calling was performed on the ChIP-seq samples using corresponding input samples with MACS v1.4.1 with a *p* value threshold of 10^−5^, and peaks were selected that were present in at least two ChIP-seq replicates (19). RUNX1 peaks identified in this way were then annotated to genes located within 50 kb on either side of the peaks (20). This analysis was performed using R (version 4.1.0) and Bioconductor (version 3.13).

### RNA polymerase II, H3K4me1, and H3K27ac ChIP-seq datasets

RNA polymerase II (RNA Pol II) binding data for resting B cells was downloaded from the Short Read Archive (accession numbers SRR955859, SRR955860, SRR955861) (21). The IgG control data for resting B cells were downloaded from Gene Expression Omnibus under accession number GSE24178 (20). Replicates were merged for downstream analysis. The ChIP-seq data with read length of 50 bp were aligned to the mouse reference genome mm9 using Bowtie (version 1.1.1). Normalized custom tracks in bigwig format were then prepared using deepTools (version 3.2.1) and visualized in UCSC genome browser.

H3K4me1 and H3K27ac ChIP-seq datasets for mouse splenocytes were downloaded from the ENCODE Consortium dataset (https://encode.org).

### Proximity ligation assay

Proximity ligation assays (PLAs) were performed with the Duolink in situ PLA kit (goat and rabbit probes; Sigma) following the manufacturer’s instructions. Images were collected by confocal microscopy using an SP5 microscope (Leica Microsystems, Wetzlar, Germany) and LAS-AF software. The following Abs were used: RUNX1 (ab23980), RUNX1 (ab35962), SRCAP (NBP145244), and SRCAP (ab9948).

### Immunofluorescence

Immunofluorescence was carried out as described by Sabbattini et al. (22).

### Western blotting

Cells were sonicated (MSE Soniprep150, 5 times 30 s on/30 s off, 14-μm amplitude), subjected to SDS-PAGE, transferred to nitrocellulose membranes, and blocked with 5% dry milk (BSA) for 1 h at room temperature. Membranes were incubated overnight with the primary Ab diluted in 0.5% milk at 4°C. They were then washed 3× for 10 min, incubated with the appropriate secondary Ab for 1 h at room temperature, washed 3× for 10 min, and developed using Millipore Crescendo ECL (Merck).

### Statistics

Statistical analysis was carried out using GraphPad Prism.

### Data availability

RNA-seq and ChIP-seq data generated for this study have been deposited at the Gene Expression Omnibus under accession GSE162704 (https://www.ncbi.nlm.nih.gov/geo/query/acc.cgi?acc=GSE162704).

## Results

### *Runx1*-knockout resting B cells are hyperresponsive to BCR stimulation

**FIGURE 1.**
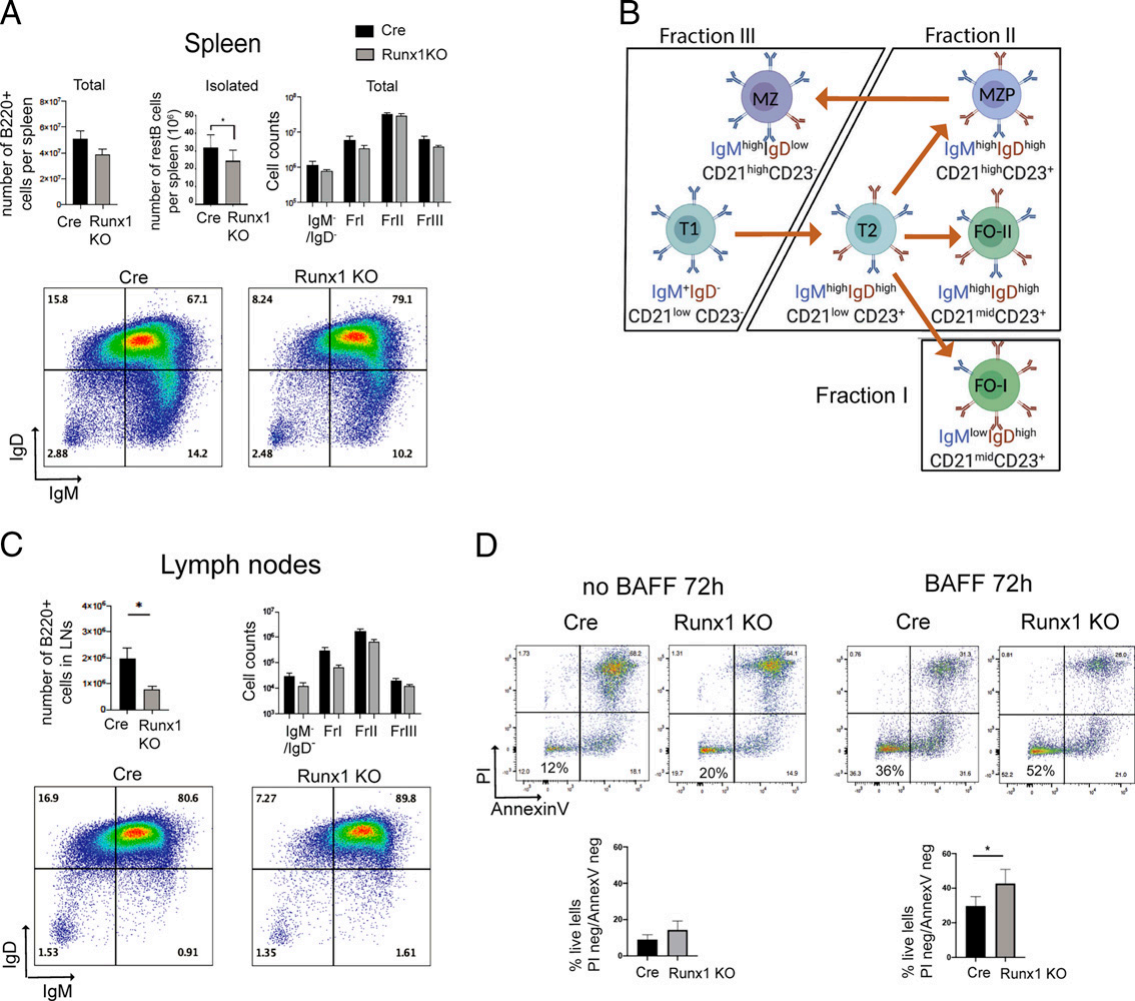
*Runx1* knockout affects responsiveness of B cells to BCR stimulation. (**A**) Top panel shows number of B220^+^ cells per spleen in CD23-cre and Runx1 c-k/o (Runx1 KO) mice (left), number of resting B cells isolated from spleens (middle), and the numbers of fraction I–III B cells and IgD^−^IgM^−^ cells (right) in the spleens of Cd23-Cre and Runx1 c-k/o mice. Bottom panel, FACS plot for splenic B220^+^ B cells showing staining for surface IgM and IgD expression. Numbers show fractions of cells based on gating for levels of IgM and IgD. Plots are representative of three Runx1 c-k/o and three control CD23-Cre spleen analyses. Percentages of total live cells for each gate are shown. (**B**) Schematic representation of the cells in fractions I–III (23, 24). (**C**) Top left panel, The percentage of B220^+^ B cells in cervical lymph nodes. Top right panel, Mean numbers of fraction I–III B220^+^ B cells in lymph nodes. Bottom panel, FACS plot of lymph node B220^+^ B cells gated according to levels of IgM and IgD surface expression. (**D**) Effect of BAFF on survival of Cre and *Runx1* c-k/o resting B cells. The cells were incubated with and without BAFF for 72 h (see *Materials and Methods*), and levels of apoptosis were assessed by staining for Annexin V and PI. Top panels show representative FACS plots, and bottom panels show numbers of cells that were negative for both markers. For (**A**), (**C**), and (**D**), values are mean ± SEM. *p ≤ 0.05, Student *t* test, *n* = 3. FO-I, type I follicular; FO-II, type II follicular; MZP, MZ precursor; T1, transitional type 1; T2, transitional type 2.

Mice that had the *Runx1* gene conditionally in activated in resting B cells (*Runx1* c-k/o) were generated by crossing a CD23-cre transgene (17) with a *Runx1* allele that has intronic LoxP sites flanking exon 4 of *Runx1* (16) (see *Materials and Methods*). The *Runx1* c-k/o mice lacked RUNX1 protein at the mature resting B cell stage (Supplemental Fig. 1A). Control CD23-Cre-only mice (henceforth referred to as “Cre-only”) expressed RUNX1 in resting B cells and showed a gradual reduction in RUNX1 levels after activation with anti-IgM + IL-4 (Supplemental Fig. 1A). The expression profile of the Runx paralogue, RUNX3, which is upregulated after 18 h of B cell activation, was unchanged in the knockout B cells (Supplemental Fig. 1B). The *Runx1* c-k/o mice had spleens and lymph nodes of apparently normal size, but FACS analysis of the pan-B cell marker B220 showed reductions in the number of B cells in spleen (Fig. 1A, top left panel) and in lymph nodes (Fig. 1C, top left panel). A similar reduction was observed in the number of resting B cells that could be isolated from the Runx1 c-k/o spleens (Fig. 1A, top middle panel).

To assess the relative proportions of B cell subpopulations in the *Runx1* c-k/o mice, we stained total splenocyte and cervical lymph node cell populations for B220, IgM, IgD, CD23, and CD21, and we analyzed staining profiles by FACS. B cells were identified by gating for B220 staining, and the relative proportions of IgD^high^/IgM^low^ (fraction I), IgD^high^/IgM^high^ (fraction II), and IgD^low^/IgM^high^ (fraction III) (23, 24) (Fig. 1B) were measured (Fig. 1A, 1C, top right and bottom left and right panels). T (CD23 ^low^/CD21^low^), follicular (FO; CD23^high^/CD21^midhigh^), and marginal zone (MZ; CD23^low^/CD21^high^) B cells were also identified among the B220^+^ cells from spleen on the basis of CD23 and CD21 staining (Supplemental Fig. 1C, left panel) and based on gating on CD21 and CD23 in total spleen cell populations (Supplemental Fig. 1D). The analysis showed that there was relatively little change in the proportions of fractions I, II, and III in the spleens of *Runx1* c-k/o mice (Fig. 1A, 1B). However, a trend toward increased numbers of MZ cells was observed in the knockout spleens (Supplemental Fig. 1C, left panel, and 1D). Increased surface IgD was also observed on MZ cells (Supplemental Fig. 1C, right panel). We conclude from these results that B cell numbers were reduced in the Runx1 c-k/o mice, but the relative proportions of the B cell subsets were not substantially altered.

CD21 is a component of the BCR costimulator complex, and a significant increase in CD21 surface levels was identified in the Runx1 c-k/o mice on B cells (B220^+^) in spleen and lymph nodes (Supplemental Fig. 1E, 1F). In the spleen, this increase was observed for fraction II, but not for fractions I and III (Supplemental Fig. 1G, 1H). It is likely that the significant increase in the number of CD21^high^ B cells in fraction II is caused by an overall increase in the expression of CD21 in FO B cells, rather than being caused by the relatively small increase that was observed in the proportion of MZ cells. This conclusion is supported by the observed increase in CD21 surface levels on the B cells in lymph nodes, which contain FO cells but lack MZ and MZ precursor cells (Supplemental Fig. 1F).

The lower overall number of B cells in the *Runx1* c-k/o spleens could be a result of reduced survival of the naive mature B cells. However, splenic resting B cells isolated from the *Runx1* c-k/o mice showed increased survival after 72-h incubation with BAFF compared with Cre-only cells (Fig. 1D). The results indicate that survival of naive B cells is enhanced by the Runx1 c-k/o. Another possible explanation for the reduced number of B cells could be that the mature splenic B cell pool is depleted by increased responsiveness to antigenic stimulation (25). We therefore set out to investigate whether the *Runx1* c-k/o B cells have an altered response to activating stimuli.

**FIGURE 2.**
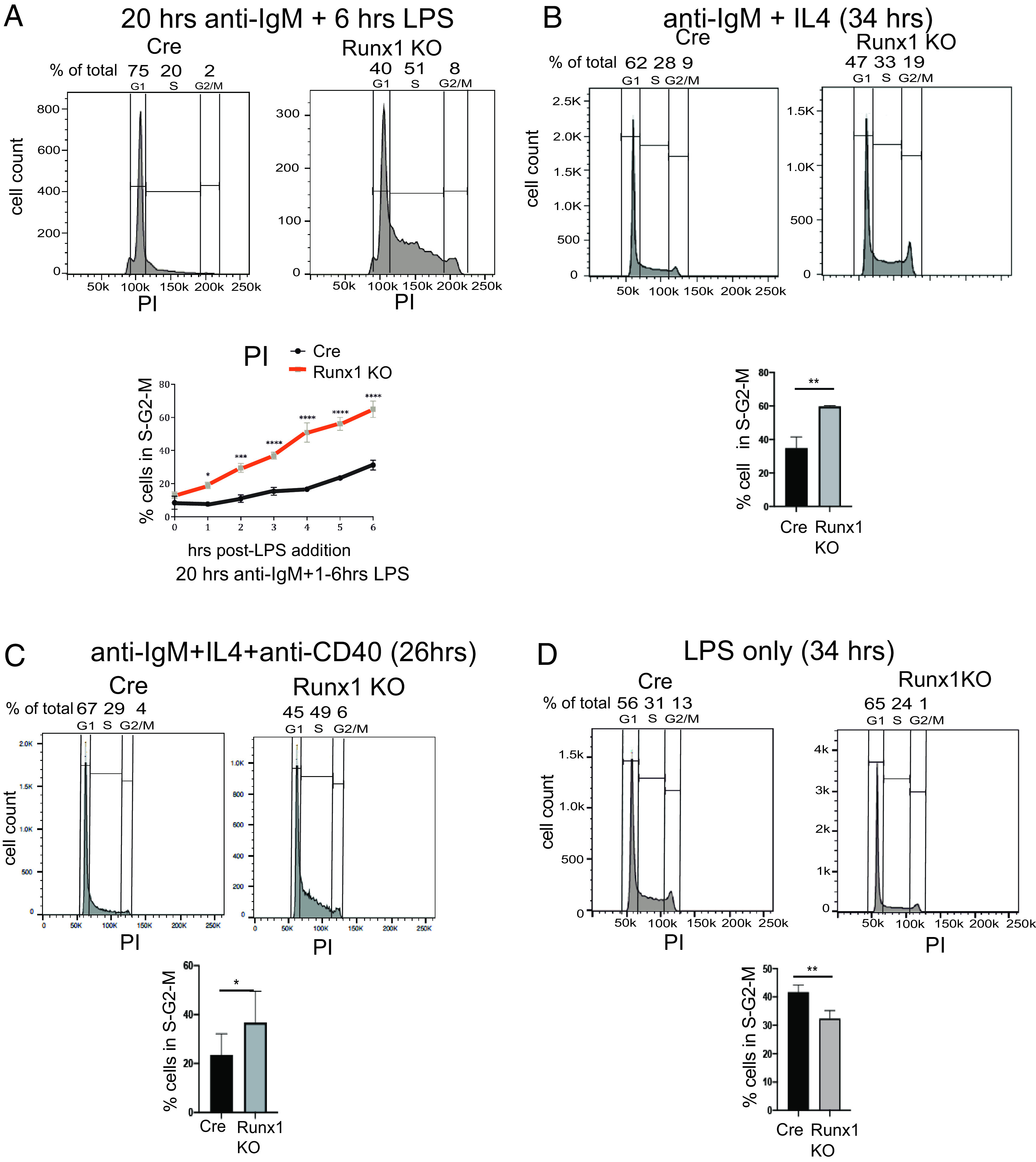
Accelerated entry of *Runx1* knockout resting B cells into S-phase in response to BCR stimulation. (**A**) Top panel, Representative FACS analysis of PI-stained Cre-only and Runx1 c-k/o B cells after 20-h treatment with anti-IgM followed by treatment with LPS for 6 h. Bottom panel, Plot showing percentage of cells in S/G_2_/M after 20-h stimulation with anti-IgM followed by 0–6 h of LPS treatment. Cells were fixed, and DNA content was measured by PI staining and FACS analysis. (**B** and **C**) Effect of the *Runx1* knockout on S-phase entry of resting B cells after stimulation of control CD23-cre (Cre) and *Runx1* c-k/o resting B cells with anti-IgM + IL-4 for 34 h (B) and costimulation with anti-IgM + IL-4 + anti-CD40 for 26 h (C). Proportions of cells in G1/S/G_2_-M were measured by PI staining as in (Fig. 1D. Top panels show representative FACS analyses. (**D**) Top panel, Representative FACS analysis carried out as in (A) on PI-stained Cre and Runx1 c-k/o B cells that had been incubated with LPS only for 34 h. Bottom panel, Percentage of cells in S/G_2_/M. (A–D, bottom panels) Mean ± SEM. *p ≤ 0.5, **p ≤ 0.01, ***p ≤ 0.001, ****p ≤ 0.0001, Student *t* test. *n* = 3.

Incubation of resting B cells with anti-IgM has been shown to stimulate the BCR response (26), resulting in entry of the cells into G_1_ within 24 h of the start of the incubation, with entry into S-phase occurring by 48 h (27, 28). Treatment of resting B cells with LPS alone has been reported to result in a limited activation response by the FO mature B cells that make up 95% of the resting B cell population, because of FO mature resting B cells having a limited capacity to respond to TLR stimulation (27). However, cells that have been prestimulated with anti-IgM respond rapidly to LPS stimulation (29). This allowed us to induce a controlled progression of purified Cre-only and *Runx1* c-k/o resting B cells to the G_1_/S transition stage of the cell cycle by incubating the cells for 20 h with anti-IgM + IL-4 followed by a time course of LPS treatment for 1–6 h. Staining of the cells with PI (Fig. 2A) showed a mean increase of ∼3-fold, from 15 to 45%, in the proportion of *Runx1* c-k/o B cells that had progressed into S-phase compared with Cre-only cells after 4 h of LPS stimulation and a 2-fold increase from 30 to 60% after 6 h of stimulation (Fig. 2A). These results indicate that the *Runx1* c-k/o resting B cells were in a hyperresponsive state after stimulation of the BCR with anti-IgM + IL-4 that caused them to respond rapidly to TLR stimulation with LPS. In contrast, Cre-only resting B cells showed a significantly slower response to stimulation under the same conditions.

To further characterize the effect of the *Runx1* knockout on cell cycle entry, we incubated the cells for longer periods with a number of different activating agents. Treatment with anti-IgM alone or with anti-IgM + IL-4 for 34 h resulted in significantly increased numbers of cells in S/G_2_/M, but with reduced numbers of cells overall in the anti-IgM alone incubation because of the increased cell survival in the presence of IL-4 (Fig. 2B, Supplemental Fig. 2A). We also tested the effect of costimulation of *Runx1* c-k/o resting B cells with anti-IgM + IL-4 and anti-CD40. After 26 h of costimulation, the mean percentage of cells in S/G_2_/M was increased from 24 to 37% (Fig. 2C). This result provides evidence that RUNX1 acts to downregulate naive B cell responses that occur through the BCR under conditions of T cell–mediated costimulation. In contrast with their hyperresponsiveness to anti-IgM and to costimulation with anti-IgM and anti-CD40, Runx1 c-k/o B cells were significantly less responsive to stimulation for 34 h with LPS alone than the Cre-only cells (Fig. 2D). Our results imply that RUNX1 downregulates BCR-mediated activation of FO B cells, altering the balance of activation via the BCR and the TLRs.

### RUNX1 antagonizes BCR-mediated upregulation of genes that affect cell cycle progression

**FIGURE 3.**
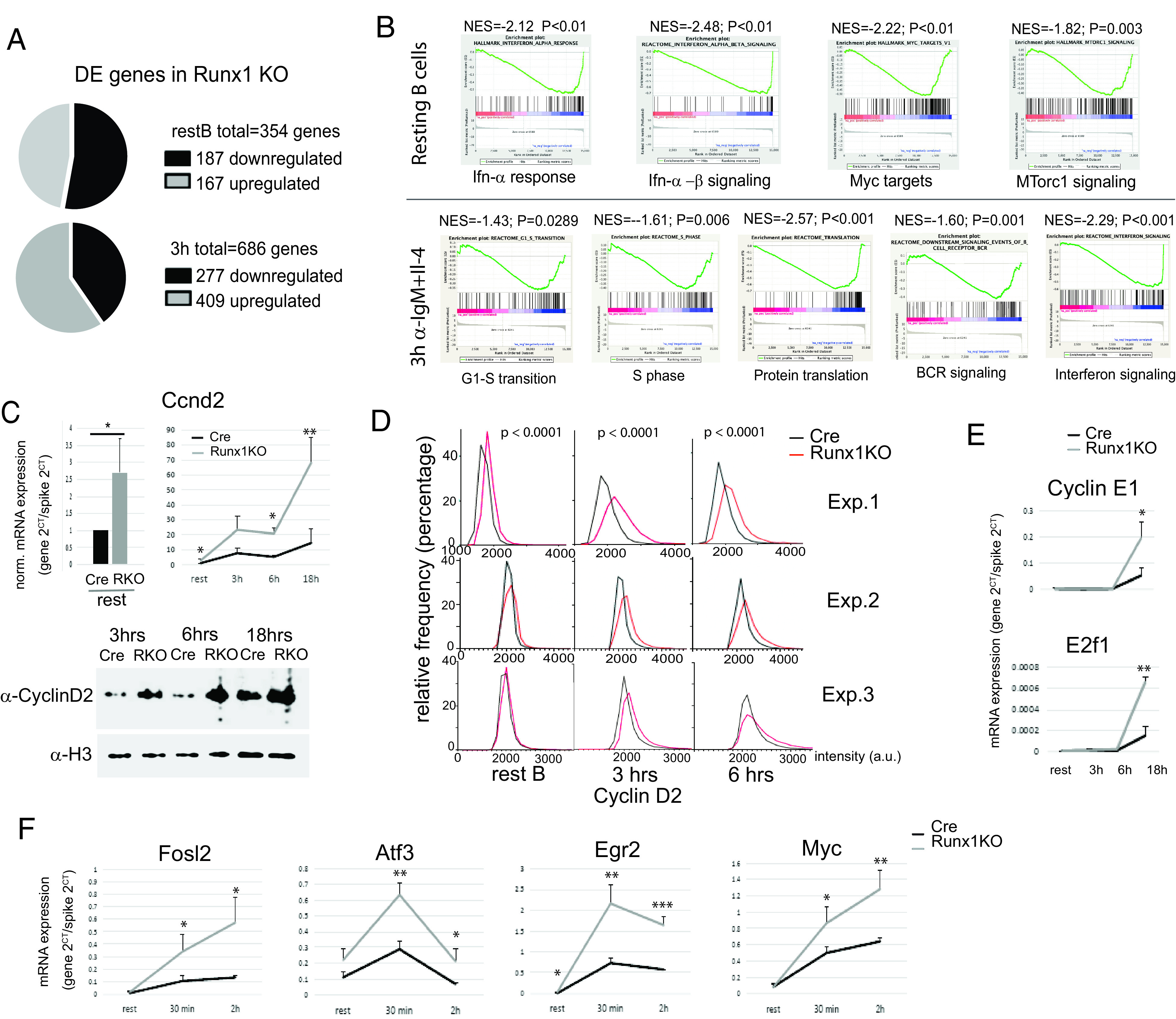
Expression of cell cycle genes during BCR-mediated activation of *Runx1* knockout B cells. (**A**) RNA-seq analysis of Runx1 c-k/o B cells showing upregulation and downregulation of genes in resting B cells (upper pie chart) and in 3-h anti-IgM activated B cells (lower pie chart) with respect to Cre-only control B cells. Three biological replicates were analyzed for each cell type. (**B**) GSEA of the RNA-seq data from 0- and 3-h anti-IgM + IL-4 activation of Runx1 c-k/o and Cre-only resting B cells. Values <0 in the graphs refer to genes whose expression is elevated in the Runx1 c-k/o B cells. (**C**) RT-qPCR analysis of expression of the *Ccnd2* gene in resting B cells (top left panel) and after activation for the indicated times with anti-IgM + IL-4 (top right panel). Bottom panel, Western blot analysis of cyclin D2 protein levels after anti-IgM + IL-4 activation for the indicated times. Loading control: histone H3. (**D**) Single-cell analysis of the level of cyclin D2 protein in resting B cells and after 3 and 6 h of anti-IgM + IL-4 treatment using quantitative, automated single-cell imaging analysis (see *Materials and Methods*). Three independent experiments are shown. *p* values obtained using the Mann–Whitney *U* test are shown above each time point. (**E** and **F**) RT-qPCR analysis of cell cycle gene transcription (**E**) and immediate early gene transcription (**F**) in the Runx1 c-k/o B cells on activation with anti-IgM + IL-4 for 0–18 h. For (C), (**E**), and (**F**), values are mean ± SD. *p ≤ 0.5, **p ≤ 0.01, ***p ≤ 0.001, Student *t* test, *n* = 3. NES, normalized enrichment score; P, nominal *p* value.

To investigate the reasons for the enhanced responsiveness of the *Runx1* c-k/o resting B cells to BCR stimulation, we used RNA-seq to analyze global gene expression patterns in *Runx1* c-k/o and Cre-only B cells at the resting B cells stage and after 3-h incubation with anti-IgM + IL-4. The analysis showed that a total of 167 genes were upregulated and 187 genes were downregulated in the Runx1 c-k/o resting B cells, and 409 genes were upregulated and 277 genes downregulated in the Runx1 c-k/o B cells after 3 h of stimulation with anti-IgM + IL-4 (adjusted *p* < 0.05) (Fig. 3A, Supplemental Fig. 2C, Supplemental Tables I and II). GSEA revealed that there was upregulation of genes associated with G_1_-S transition, S-phase, BCR signaling, and protein translation functions at 3 h after anti-IgM/IL-4 addition in the *Runx1* c-k/o B cells compared with the level observed in Cre-only cells (Fig. 3B, Supplemental Fig. 2D). The GSEA also revealed upregulation of signaling and immune response genes at both 0 and 3 h after anti-IgM/IL-4 addition and downregulation of late cell cycle G_2_-M and mitotic spindle genes (Supplemental Fig. 2D).

To gain additional insights into the hyperresponsive phenotype of the *Runx1* c-k/o B cells, we carried out a more detailed analysis of changes in the expression of genes that are involved in regulating entry of B cells into the cell cycle. Cyclin D2 is the main cyclin D involved in BCR-mediated activation as indicated by the failure of cyclin D2–deficient B cells to proliferate efficiently in response to stimulation of the BCR (30, 31). We analyzed the kinetics of cyclin D2 (*Ccnd2*) expression in isolated *Runx1* c-k/o resting B cells upon activation with anti-IgM + IL-4 during a time course from 0 to 18 h. RT-qPCR analysis of *Ccnd2* mRNA showed a large increase in *Ccnd2* mRNA levels in the knockout after 3-h treatment with anti-IgM + IL-4 and continuing through to 18 h (Fig. 3C, top panel).

The increase in cyclin D2 expression was confirmed by western blotting, which showed substantial increases in the level of cyclin D2 protein in *Runx1* c-k/o cells relative to Cre-only cells at 3, 6, and 18 h after addition of anti-IgM + IL-4 (Fig. 3C, bottom panel). The analysis of Cyclin D2 protein was further extended by using high-content single-cell microscopy followed by automated quantitative image analysis to measure the levels of cyclin D2 in the nuclei of individual cells (Fig. 3D). The results of this analysis showed a significant increase in cyclin D2 levels in *Runx1* c-k/o resting B cells and after 3 and 6 h of anti-IgM + IL-4 treatment (see *Materials and Methods* for details). The analysis also showed that the increase is unimodal, with the effect distributed across the entire cell population rather than being confined to a subset of cells (Fig. 3D). These data indicate that the presence of RUNX1 in resting B cells reduces *Ccnd2* transcription and is associated with a lower rate of increase in the level of *Ccnd2* mRNA and protein as the cell progresses through G_1_. BCR-mediated activation of naive B cells is strongly dependent on cyclin D2, whereas LPS is known to activate naive B cells independently of cyclin D2 and instead uses cyclin D3 (30, 31). This could explain the difference in the effect of the *Runx1* knockout on activation by anti-IgM + IL-4 compared with LPS only (Fig. 2B, 2D).

In contrast with the effect of the *Runx1* knockout on cyclin D2 expression, levels of transcription of the gene encoding cyclin E1 (*Ccne1*) were unchanged in Runx1 c-k/o resting B cells and after 3- and 6-h incubation with anti-IgM + IL-4 compared with Cre-only B cells (Fig. 3E). Increased levels of *Ccne1* and *E2f1* mRNA were observed only after 18 h of anti-IgM + IL-4 stimulation. This is consistent with previous reports that *Ccne1* transcription is upregulated in late G_1_ by the E2F factors (32). *E2f1* transcription has been shown to be subject to autoactivation by E2F factors that have been activated by Rb phosphorylation (33).

We also examined the activation profile of several immediate early genes in response to anti-IgM + IL-4 stimulation. Levels of transcription of the *Fosl2*, *Atf3*, *Egr2*, and *Myc* genes were analyzed at time points from 0 to 2 h after addition of anti-IgM. Expression of *Atf3* and *Egr2* peaked at 30 min in Cre-only cells and then declined. This profile was maintained in the *Runx1* c-k/o B cells, but the expression peak at 30 min was strongly enhanced, and overall expression was increased at all three time points (Fig. 3F). Expression of *Fosl2* and *Myc* showed a progressive increase at 30 min and 2 h in Cre-only cells, and the slope of the expression curve was shifted upward for both genes in the *Runx1* c-k/o cells (Fig. 3F). These results implicate *Runx1* in the downregulation of cell cycle and immediate early genes during BCR-mediated activation of naive resting B cells.

### RUNX1 binds to poised cell cycle and immediate early genes in resting B cells

**FIGURE 4.**
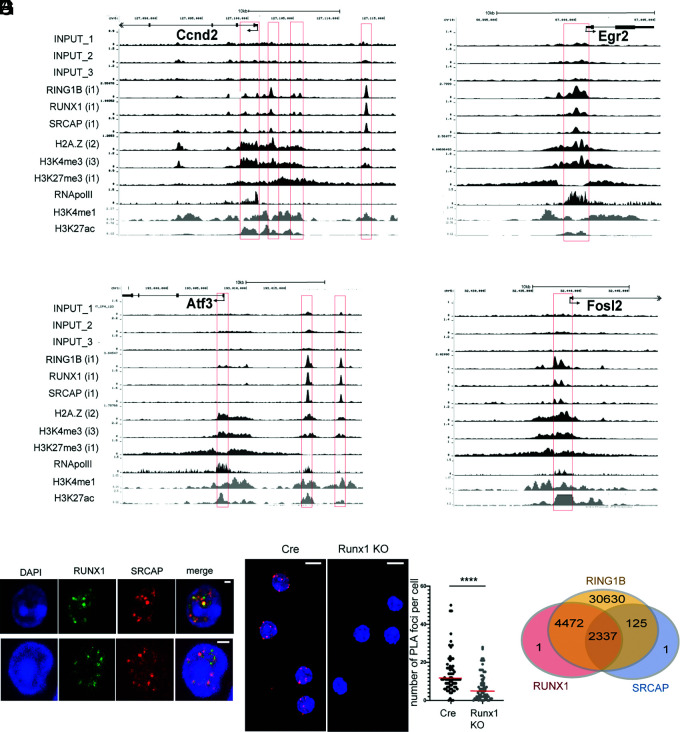
ChIP-seq analysis of cell cycle and immediate early genes. (**A**–**D**) Tracks shown in black represent ChIP-seq profiles from purified resting B cells for *Ccnd2* (A), *Egr2* (B), *Atf3* (C), and *Fosl2* (D). Gray tracks represent data from splenocytes downloaded from ENCODE. The data for RNA pol II were downloaded from a published dataset obtained by analysis of purified resting B cells (21) (see *Materials and Methods*). The inputs for each track are indicated by numbering of the input tracks with the relevant input number shown in brackets for each analytical track. Red boxes highlight the positions of TSSs and the position of candidate enhancers co-occupied by RUNX1, SRCAP, and RING1B. (**E**) Localization of RUNX1 and SRCAP was determined by immunofluorescence analysis of resting B cells. Top and bottom panels show different magnifications of single cells. Scale bars, 1 μm. (**F**) The PLA was carried out on Cre-only resting B cells (Cre) using Abs specific for RUNX1 (Ab A) and for SRCAP (Ab B). To control for nonspecific interactions, we also carried out the PLA on *Runx1* c-k/o B cells. The control cells showed some background foci, predominantly in association with the nuclear envelope and with a much lower signal intensity than was observed in the Cre cells. Digital analysis used to screen out foci that were in contact with the nuclear envelope in the Cre and *Runx1* c-k/o B cells (see *Materials and Methods*) showed that there were significantly fewer foci per cell in the knockout B cells (right panel). Red, PLA amplification signals; blue, Hoechst dye staining the nuclei. Scale bars, 5 μm. Unpaired *t* test, ****p ≤ 0.0001. (**G**) Venn diagram summarizing the degree of overlap of peaks by ChIP-seq peak calling for binding of RUNX1, SRCAP, and RING1B in resting B cells.

ChIP-seq was carried out on chromatin from wild-type resting splenic B cells obtained from C57Bl6 mice to examine profiles of factor binding and epigenetic modification at genes that showed altered expression in the *Runx1* c-k/o B cells (see *Materials and Methods* for details). RUNX1 binding peaks were found to be widely distributed at promoters and putative enhancers of the affected genes (Fig. 4A–D). The *Ccnd2* and *Atf3* genes had strong peaks of RUNX1 binding at distally located elements that had the epigenetic characteristics of enhancer or silencer sequences (Fig. 4A, 4C). Strong binding peaks for RUNX1 were located close to the transcription start site (TSS) of the *Egr2* and *Fosl2* genes (Fig. 4B, 4D) in regions with epigenetic profiles that are indicative of overlapping promoter/enhancer sequences (34). Overall, these results suggest that RUNX1 binds to promoters and enhancers at cell cycle and immediate early genes in resting B cells.

RUNX1 has the characteristics of a pioneer factor that can initiate binding to closed nucleosomal chromatin (35) and has also been shown to interact with the Swi/Snf-like Brg1/Brm-associated factor (BAF) chromatin remodeling complex in thymocytes (36). Our initial ChIP analysis of chromatin remodeling subunits indicated that components of another remodeling complex, the SRCAP complex, bind to active genes in resting B cells and to genes that are inducible by activation. ChIP-seq analysis of the SRCAP protein revealed that binding peaks for SRCAP colocalized with RUNX1 at poised and active genes (Fig. 4A–D). The SRCAP nucleosome remodeling complex is responsible for incorporating histone H2A.Z into nucleosomes (37), and our analysis showed that H2A.Z is enriched at active and poised promoters in resting B cells (Fig. 4A–D).

The frequent overlap of RUNX1 and SRCAP binding that we observed at hyperresponsive genes in resting B cells suggests that RUNX1 might be interacting with SRCAP and recruiting it to poised genes. The very large size of the SRCAP protein (360 kDa) makes it challenging to carry out coimmunoprecipitation experiments. Therefore, we used immunostaining of SRCAP and RUNX1 to examine localization of the two proteins. The results show strong staining foci for both proteins with overlap observed between the majority of the foci (Fig. 4E). PLAs performed with Abs against RUNX1 and SRCAP and using Runx1 c-k/o resting B cells as a negative control confirmed that there is close contact between RUNX1 and SRCAP in the resting B cells (Fig. 4F). This is consistent with the genome-wide colocalization of SRCAP binding with binding peaks for RUNX1 (Fig. 4G).

The ChIP-seq analysis also revealed that a number of the hyperresponsive cell cycle and immediate early genes identified in the expression analysis had a poised epigenetic profile in wild-type resting B cells. The term “gene poising” describes a state where promoters are held in an open state, ready for rapid action in response to appropriate signals. Gene poising was first described in *Drosophila* (38) and has also been shown to occur in embryonic stem (ES) cells (39) and in a variety of other cell types, including resting CD4^+^ T cells (40), memory T cells (41), differentiated neurons (42), and thymic epithelial cells (43). The promoter-proximal regions of the poised genes had broad regions of enrichment for histone H3K27me3 (Fig. 4A–D), which is deposited by the polycomb PRC2 complex member EZH2 and is considered to be a repressive mark. Enrichment for H3K27me3 is also associated with poised promoters in ES cells, CD4^+^ T cells, neurons, and ES cells, where it forms part of the bivalent H3K4me3/H3K27me3 epigenetic mark (40, 42, 44, 45, and reviewed in Ref. 46). The regions of H3K27me3 enrichment that we observed at the hyperresponsive cell cycle and immediate early genes in resting B cells showed partial overlap with regions of enrichment for H3K4me3, indicating that these promoters are marked with this bivalent epigenetic signature (Fig. 4A–D).

RING1B, which is the catalytically active component of the polycomb PRC1 complex (47), has been shown to bind to poised and active genes in a variety of cell types (39, 48–51), and RUNX1 and RING1B have been reported to co-occupy binding regions in megakaryocytes and T cells (51). Our ChIP-seq analysis showed strong colocalization of binding of RUNX1 and RING1B in resting B cells (Fig. 4A–D, 4G). The overlapping RUNX1 and RING1B peaks were associated with distally located sequences that had the epigenetic signature of enhancers at the *Ccnd2* and *Atf3* genes and were also found at or close to promoters of genes that were hyperresponsive in *Runx1* c-k/o B cells (Fig. 4A–D). The colocalized peaks of RUNX1 and RING1B binding in resting B cells did not show any specific colocalization with the regions of enrichment for H3K27me3, similar to our previous observation that RING1B binds at active genes in resting B cells, independently of H3K27me3 (48).

Genome-wide analysis of peaks of RUNX1 binding showed that 60% of the DEGs identified by the RNA-seq analysis had peaks located within a region extending from −50 to +50 kb relative to the gene (Supplemental Table III; see *Materials and Methods*), with a roughly equal division between negatively and positively regulated genes in this group. Protein interaction and Gene Ontology analysis revealed that the DEGs that were proximal to RUNX1 peaks were enriched for response to stimulus and for negative regulation of the immune response. This finding further supports the conclusion that RUNX1 is involved in regulating the humoral immune response. The major Gene Ontology category identified for the nonproximal DEGs was “binding to nucleotides,” indicating that these genes form a group that is functionally separate from the proximal genes and may be subject to secondary regulation. Similar to previous observations for the B cell factor PAX5 (20), we found that <5% of the 8812 genes that have RUNX1 peaks located within 50 kb of the gene have their expression affected by the *Runx1* knockout. Likely reasons for the large number of unaffected genes include a requirement for additional transcription factors and cofactors and/or the action of epigenetic and factor-mediated repression of target gene promoters in B cells.

To examine RNA Pol II binding at poised promoters, we downloaded published ChIP-seq data for RNA Pol II in resting mouse B cells (21). The ChIP-seq tracks show high levels of RNA Pol II in the regions around the promoters of the *Ccnd2*, *Egr2*, *Atf3*, and *Fosl2* genes compared with the levels of Pol II in the gene bodies (Fig. 4A–D, Supplemental Fig. 3C). The presence of higher levels of Pol II at promoter regions compared with the levels observed in the body of the gene has been shown to be a characteristic feature of poised genes in *Drosophila* embryonic cells (52) and mouse ES cells (53).

### Knockout of Runx1 reduces binding of SRCAP and increases binding of BRG1 and RING1B at the *Ccnd2* gene

**FIGURE 5.**
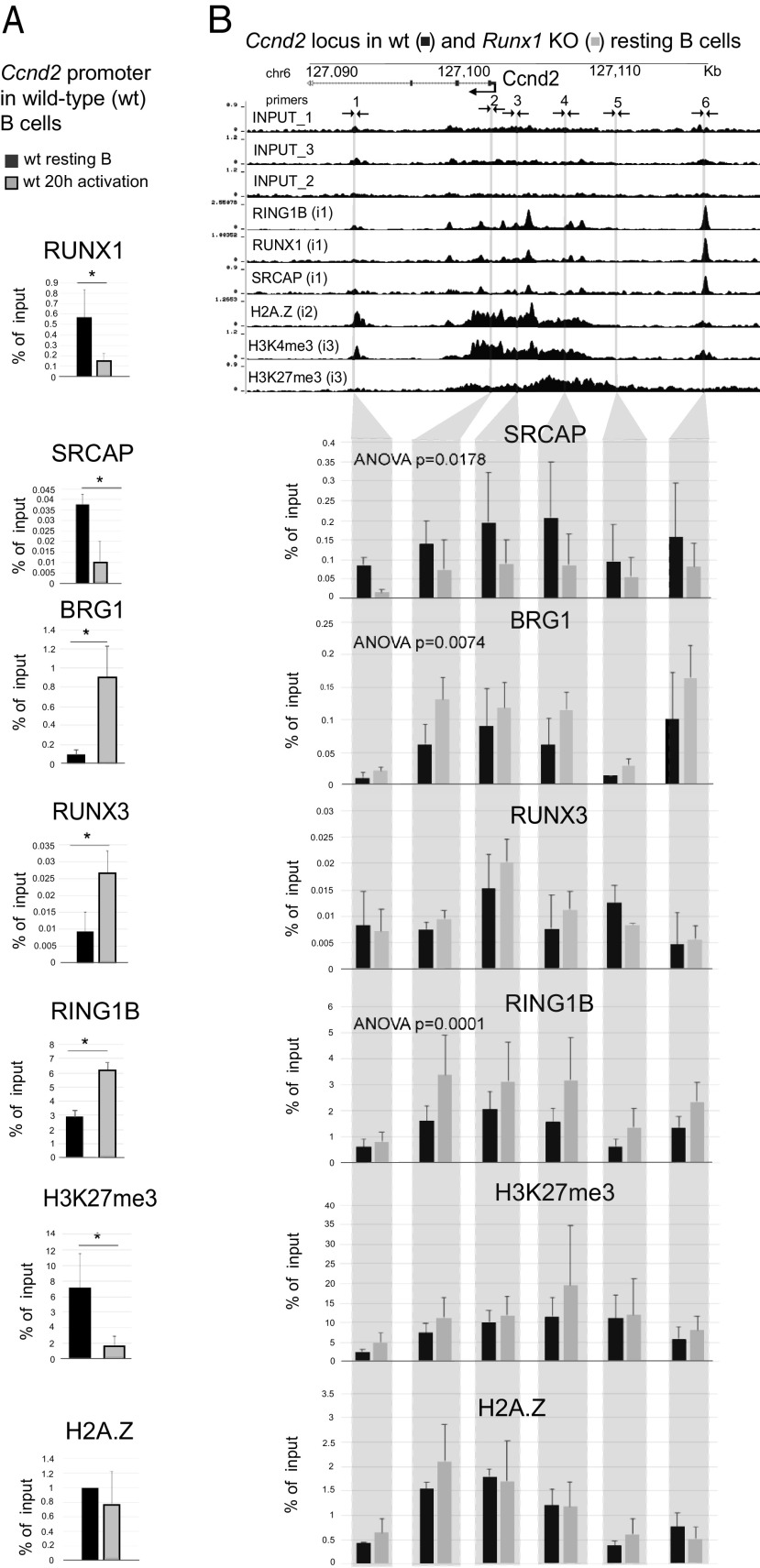
Analysis of the epigenetic architecture of the *Ccnd2* promoter in B cells. (**A**) ChIP-qPCR of the indicated proteins and histone modifications at the *Ccnd2* promoter in wild-type (wt) B cells at the resting stage (black) and after 20-h activation with anti-IgM + IL-4 (gray). Values are mean ± SD, *n* = 3. *p ≤ 0.5, **p ≤ 0.01, Student *t* test. (**B**) ChIP-qPCR at six DNA sites corresponding to the *Ccnd2* promoter and surrounding regions in Cre-only (black) and Runx1 c-k/o (gray) resting B cells. Vertical lines show positions of primer pairs used for the ChIP analysis. Mean ± SD, *n* = 3; *p* values were calculated by ANOVA.

To further analyze the role of RUNX1 in configuring the epigenetic profile of poised cell cycle genes in resting B cells, we focused on the promoter and upstream region of the *Ccnd2* gene. Cyclin D2 has critical roles in B cell activation and also has been shown to be positively regulated by the *RUNX1*-ETO fusion oncoprotein in acute myeloid leukemia (54). We first carried out an analysis of enrichment of H3K27me3 and H2A.Z and binding of SRCAP, BRG1, RING1B, and the RUNX1 paralog RUNX3 at the *Ccnd2* promoter in wild-type resting B cells and after 20-h treatment with anti-IgM + IL-4. The results of the analysis, which are shown in (Fig. 5A, revealed that BCR stimulation of wild-type cells resulted in a statistically significant reduction in binding of RUNX1 and SRCAP and significantly increased binding of BRG1 and RUNX3. Binding of the PRC1 component, RING1B, was significantly increased, and enrichment for the PRC2 marker, H3K27me3, was strongly reduced in the activated cells, whereas H2A.Z was largely unchanged. The fact that RUNX3 showed a strong increase in binding during BCR activation, which coincided with decreased binding of RUNX1 (Fig. 5A), suggests that there is a switch between the two Runt-related factors. This is supported by the observation that the level of RUNX1 protein declines on activation of the resting B cells (Supplemental Fig. 1A), while RUNX3 protein is upregulated (Supplemental Fig. 1B; see also Ref. 55). The large increase in binding of BRG1 when the cells are activated (Fig. 5A) suggests that there is also a switch from binding of the SRCAP remodeling complex to binding of the BRG1-containing switch/SNF (SWI/SNF) complex at the *Ccnd2* promoter during B cell activation.

**FIGURE 6.**
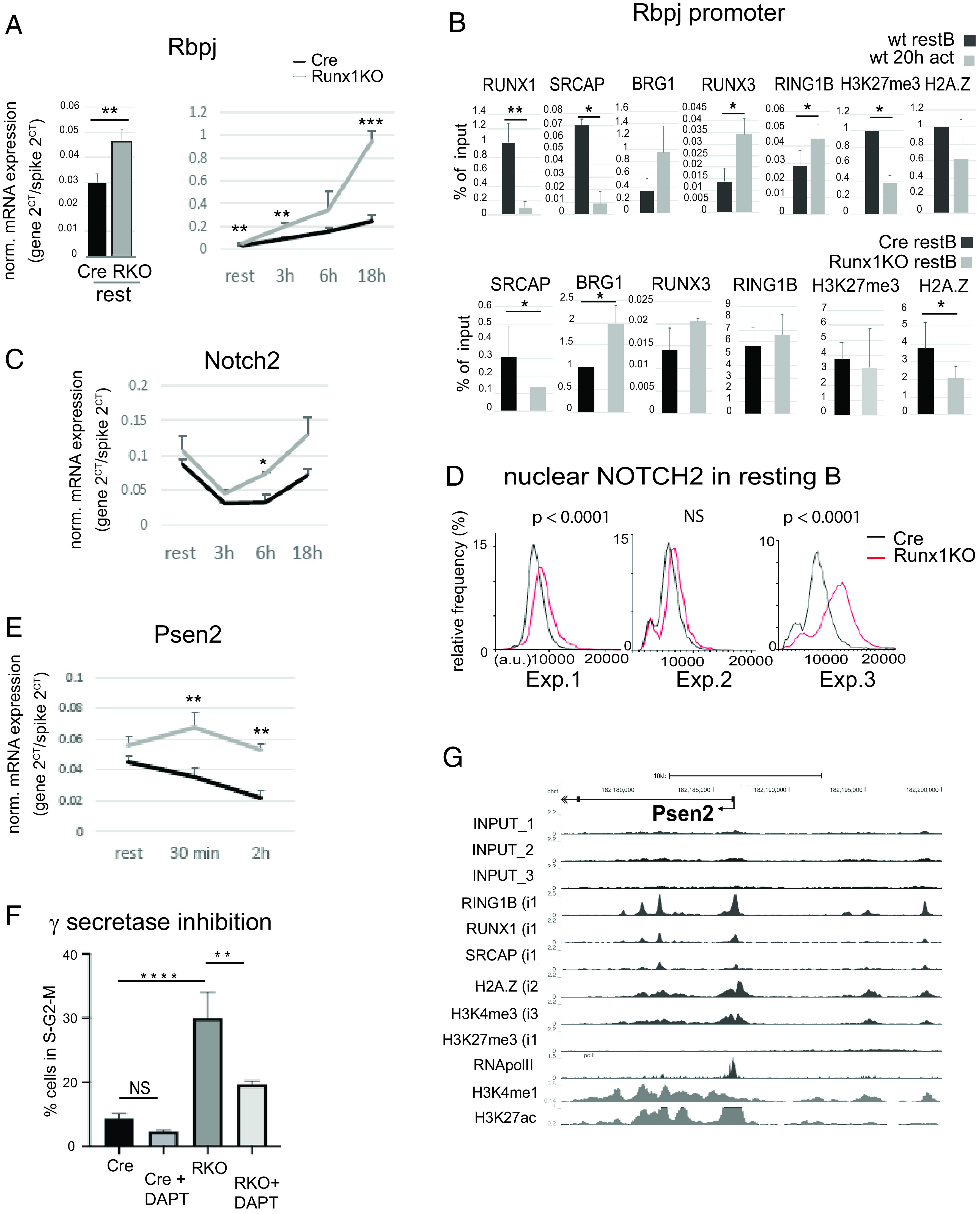
Runx1 represses Notch signaling in B cells. (**A**) RT-qPCR analysis of *Rbpj* expression in CD23-cre and Runx1 c-k/o resting B cells (left panel) and on activation with anti-IgM + IL-4 for 0–18 h (right panel). (**B**) Top panel, ChIP-qPCR of factor binding and histone modifications at the *Rbpj* promoter in wild-type (wt) resting B cells and after 20-h anti-IgM + IL-4 activation. Bottom panel, The same primers were used to analyze Cre-only (black) and Runx1 c-k/o (gray) resting B cells. (**C**) RT-qPCR of Notch2 transcript levels during a B cell activation time course as in (A). (**D**) Single-cell analysis showing the level of NOTCH2 protein in the nuclei of resting B cells (see *Materials and Methods*). The results of three independent experiments (Exp. 1–3) are shown. *p* values obtained using the Mann–Whitney *U* test for each experiment are shown above the plots. (**E**) RT-qPCR of transcripts from *Psen2* after 30-min and 2-h activation of Runx1 resting B cells with anti-IgM + IL-4. (**F**) Analysis of the effect of the γ-secretase inhibitor DAPT on entry of resting B cells into S-phase in response to 18-h incubation with anti-IgM + IL-4 followed by 6-h treatment with LPS. Values shown represent mean ± SD. Significance was calculated using one-way ANOVA followed by Tukey’s multiple comparison test. (**G**) ChIP-seq analysis of the promoter and upstream region of the *Psen2* gene. Details of the individual ChIP-seq tracks are as described in the legend to (Fig. 4. (A, C, and E) Values are mean ± SD. *p ≤ 0.5, **p ≤ 0.01, ***p ≤ 0.001, ****p ≤ 0.0001. Student *t* test; *n* ≥ 3. NS, nonsignificant.

To analyze the effect of the *Runx1* knockout on factor binding and epigenetic marks across the regions upstream and downstream from the *Ccnd2* TSS, we used a series of qPCR-ChIP primers that covered the *Ccnd2* promoter region, as well as candidate upstream and downstream regulatory elements and the domain of H3K27me3 enrichment (Fig. 5B). The results of the qPCR-ChIP carried out on Cre-only and *Runx1* c-k/o resting B cells showed that the *Runx1* knockout led to a clear and significant decrease in binding of SRCAP and a significant increase in binding of BRG1 at the promoter and upstream and downstream regions (Fig. 5B). Binding of RING1B was also significantly increased. Binding of RUNX3 and enrichment for H3K27me3 and H2A.Z were largely unaffected by the *Runx1* knockout in resting B cells (Fig. 5B). The effect of the knockout on binding of RUNX3 and BRG1 to the *Ccnd2* and *Rbpj* promoters was also analyzed after 20 h of anti-IgM + IL-4 stimulation. Although binding of both Runx3 and BRG1 is increased in wild-type cells at 20 h post-BCR activation (Figs. 4A and (6B), the Runx1 knockout had little effect on the increase in binding of RUNX3 at this stage, whereas the level of BRG1 binding was higher in the knockout cells compared with Cre-only cells (Supplemental Fig. 3E).

Taken together, the results of the ChIP-seq and ChIP-qPCR analysis reveal binding of RUNX1, SRCAP, and RING1B accompanied by a poised bivalent epigenetic configuration and enrichment for H2A.Z at several cell cycle and immediate early genes in resting B cells. Knockout of RUNX1 reduces SRCAP binding and increases binding of the activation-associated SWI/SNF component BRG1 and binding of RING1B at the *Ccnd2* gene, suggesting that RUNX1 recruits SRCAP to enhancers and promoters but has a more complex relationship with RING1B binding. The ChIP analysis also shows that RUNX1 is largely replaced by RUNX3 at the *Ccnd2* promoter during BCR-stimulated activation of wild-type resting B cells. Although RUNX3 has been reported to interact with BRG1, recruiting it to promoters (56), the fact that RUNX3 binding was not affected by the *Runx1* knockout in BCR-stimulated cells suggests that the enhanced BRG1 binding that we observed in the knockout cells at 20 h compared with 20-h stimulated Cre-only cells (Supplemental Fig. 3E) was an epigenetic effect arising from the earlier partial switch between SRCAP and BRG1 that resulted from the absence of RUNX1 protein in resting B cells.

### Notch signaling is enhanced in hyperresponsive *Runx1* c-k/o resting B cells

Among the genes that were upregulated in the hyperresponsive *Runx1* c-k/o resting B cells were components of the Notch signaling pathway. Signaling through the transmembrane NOTCH receptors occurs when the receptors are bound by ligands belonging to the Jagged and delta-like families. This leads to cleavage and release of the extracellular domain of the receptors, followed by a second cleavage mediated by presenilin family members, which act as the catalytic subunits of the γ-secretase complex. Presenilin-mediated cleavage results in release of the Notch intracellular domain, which then translocates to the nucleus, where it binds to RBPJ, forming a complex that recruits other coactivators and upregulates promoters of target genes (reviewed in Refs. 57, 58). Notch signaling via NOTCH2 has generally been associated with specification of MZ B cells during B cell maturation, whereas development of FO B cells was left unaffected by c-k/o of *Notch2* (59, 60). However, stimulation of the Notch pathway has been shown to increase proliferation of purified splenic FO B cells in response to anti-IgM stimulation (61), suggesting that Notch signaling does have a role in regulating the dynamics of B cell activation.

Our analysis of Notch pathway genes revealed that expression of *Rbpj*, *Notch2*, and *Psen2* was altered in the *Runx1* c-k/o B cells (Fig. 6A, 6C, 6E). All three genes showed elevated expression at the resting B cell stage. *Notch2* and *Rbpj* also showed strongly elevated expression after 18 h of stimulation (Fig. 6A, 6C), whereas expression levels of *Psen2* in the knockout cells were increased in resting B cells and after 30 min of anti-IgM + IL-4 activation before declining (Fig. 6E). Epigenetic and factor binding analysis of the *Rbpj* promoter (Fig. 6B) revealed a similar profile to the *Ccnd2* gene (Fig. 5A), suggesting an overlap in regulatory mechanisms for the two genes. We also used high-content single-cell microscopy to measure the levels of NOTCH2 protein in the nuclei of Cre-only and *Runx1* c-k/o resting B cells. The results of three independent experiments show a clear trend toward increased levels of nuclear Notch2 in *Runx1* c-k/o resting B cells with highly significant differences observed in two of the three experiments (Fig. 6D). The observation that distribution of the increased levels of Notch2 in the nuclei of isolated resting B cell populations is largely unimodal indicates that the *Runx1* knockout increases the level of nuclear NOTCH2 in FO B cells. We also analyzed expression of the Notch target genes, *Myc*, *Bcl2*, and *Skp2*. All three genes showed elevated expression at different stages of G_0_/G_1_ (Supplemental Fig. 3D). In particular, *Skp2*, which has been shown to be a direct target of Notch signaling (62, 63), is strongly upregulated in late G_1_ in the *Runx1* c-k/o B cells. *Skp2* encodes a subunit of the SCF^SKP2^ E3 ubiquitin ligase, which plays a key role in promoting entry into S-phase by mediating degradation of the CDK inhibitor p27 (64).

The role of Notch signaling in the hyperresponsive phenotype was further tested by preincubating the Cre-only and *Runx1* c-k/o cells with the γ-secretase inhibitor DAPT, followed by incubation with anti-IgM + IL-4 for 18 h and LPS for 6 h. The results showed that inhibiting the Notch pathway significantly reduced the proportion of the knockout cells that entered S-phase (Fig. 6F), indicating that Notch signaling has an important role in the hyperresponsive phenotype of the *Runx1* c-k/o B cells. Consistent with Notch activation, we found that expression of CD21, which is encoded by the *Cr2* gene, is significantly upregulated in fraction II of Runx1 c-k/o resting B cells (Supplemental Fig. 1H). Fraction II is enriched for IgM^hi^ FO B cells (Fig. 1B). It has been reported previously that ectopic expression of activated NOTCH upregulates the BCR coreceptor CD21 in human B lymphoma cell lines (65).

### RUNX1 regulates transcription of genes that affect B cell functions before and during activation

**FIGURE 7.**
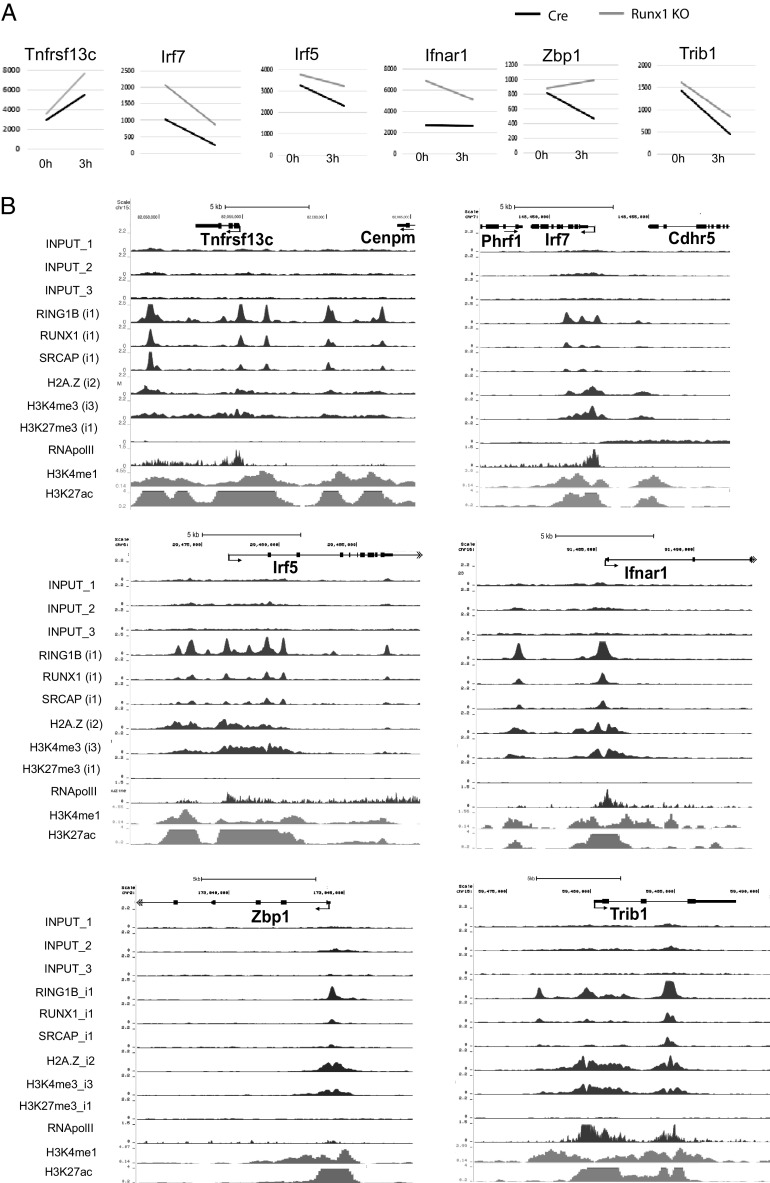
Factor binding profiles of genes that are repressed by RUNX1. (**A**) Basemean values for expression of genes with functional roles in B cells that are upregulated in the *Runx1* c-k/o B cells (see Table I). Values were obtained from the RNA-seq analysis at 0 and 3 h of anti-IgM + IL-4 treatment (see Supplemental Tables I and II). All values are the mean of three biological replicates. Adjusted *p* values for the differences in gene expression levels were <0.05 for at least one of the points. (**B**) ChIP-seq tracks showing the profiles of factor binding and histone modification in resting B cells of the genes shown in (A).

In addition to the role played by RUNX1 in regulating cell cycle genes, the RNA-seq and GSEA of *Runx1* c-k/o resting and 3-h activated B cells revealed changes in expression of a number of genes that regulate key functions in B cells. A total of 13 genes were identified that fit into this category, of which 10 were upregulated and 4 downregulated in the *Runx1* c-k/o B cells (Table I, Fig. 7A, Supplemental Fig. 3A). Examination of the ChIP-seq profiles of these genes showed strong colocalization of binding peaks for RUNX1, SRCAP, and RING1B in the regions around their promoters and putative enhancer elements (Fig. 7B, Supplemental Fig. 3B). Only one of the upregulated genes, IRF7, showed the combination of high RNA Pol II at their promoters and enrichment for H3K27me3 that is characteristic of poised promoters. The other genes had relatively low levels of promoter-bound RNA Pol II, with the exception of *Ifnar1*, which had intermediate levels, but no H3K27me3 (Fig. 7B).

**Table I.** Changes in expression of genes that affect B cell functions in *Runx1* c-k/o measured by RNA-seq analysis of resting B cells and after 3-h activation with anti-IgM + IL-4

Gene	Base Mean	Fold Change 0 h	Fold Change 3 h	Functions	References
*Ptpn22*	995	1.3 ↓	1.9 ↓	Phosphatase; regulates SRC-family kinase; negative regulator of BCR signaling and B cell apoptosis	Armitage et al. (79)
*Lrrk2*	6056	1.7 ↓	3.7 ↓	Kinase; involved in regulation of B cell homeostasis	Kubo et al. (80)
*Ccr6*	974	3.0 ↓	4.0 ↓	Chemokine receptor; expressed on naive and memory B cells	Krzysiek et al. (81), Bowman et al. (82)
*Cd22*	11,926	1.2 ↓	1.4 ↓	Inhibitory coreceptor; negative regulator of BCR signaling	O’Keefe et al. (74), Müller and Nitschke (83)
*Irf7*	1046	2.0 ↑	3.5 ↑	Regulation of IFN response gene transcription	Ning et al. (84)
*Irf5*	3126	1.1 ↑	1.4 ↑	Regulation of IFN response gene transcription	Ban et al. (85)
*Ifnar1*	4324	2.5 ↑	1.9 ↑	Subunit of IFN-α receptor	Pogue et al. (86)
*Zbp1*	787	1.1 ↑	2.1 ↑	Encodes DNA-dependent activator of IFN-regulatory factors	Takaoka et al. (87)
*Trib1*	1081	1.1 ↑	1.9 ↑	Negative regulator of Ab production	Simoni et al. (88)
*Lgals9*	1512	1.2 ↑	1.7 ↑	Negative regulator of BCR signaling	Cao et al. (66)
*Stat1*	3506	1.0 ↑	1.5 ↑	Enhancement of IFN-induced gene expression	Cheon et al. (89)
*Tnfrsf13c*	4888	1.2 ↑	1.4 ↑	Encodes BAFF receptor; promotes survival of FO and MZ B cells	Thompson et al. (90), Schiemann et al. (91)
*Bank1*	7290	1.0 ↑	1.3 ↑	Inhibition of CD40- and BCR-mediated activation of B cells	Aiba et al. (92)

Adjusted *p* values for all differences shown < 0.05 (see Supplemental Tables I and II for the full set of base mean values). Arrows indicate fold downregulation (↓) or upregulation (↑) in the *Runx1* c-k/o B cells.

Among the genes that were upregulated, *Tnfrsf13c* encodes the BAFF receptor, providing an explanation for the increased survival of the *Runx1* c-k/o resting B cells in response to incubation with BAFF (Fig. 1D). Changes were also observed in the expression of genes that affect BCR signaling. *Cd22* encodes an inhibitory coreceptor that antagonizes the BCR, and *Ptpn22* encodes a phosphatase, which is also a negative regulator of BCR signaling. Both genes were downregulated in the knockout, and the reduction in *Cd22* expression was confirmed at the protein level (Supplemental Fig. 2B). The positive regulation of both genes by RUNX1 is consistent with the idea that RUNX1 has a predominantly inhibitory effect on BCR signaling. However, upregulation of *Lgals9* in the resting and 3-h activated *Runx1* c-k/o B cells and *Bank1* in the 3-h activated knockout cells indicates that RUNX1 has complex effects on BCR functioning. *Lgals9* encodes Galectin-9, which negatively regulates BCR signaling by inhibiting microclustering of the BCR (66). The BANK protein inhibits CD40- and BCR-driven activation of B cells, and our RNA-seq results showing that *Bank1* expression is reduced by 4-fold in cre-only (wild-type) B cells after 3-h treatment with anti-IgM + IL-4 (Supplemental Table II) are consistent with previous observations that BCR activation reduces BANK expression (67). Our analysis of the *Runx1* c-k/o B cells shows that RUNX1 contributes to this downregulation. The finding that RUNX1 has a role in downregulating inhibitors of BCR activation and costimulation suggests that RUNX1 is not simply antagonizing Ag-mediated activation but is instead cooperating with the BCR to fine-tune the amplitude of the Ag response.

**FIGURE 8.**
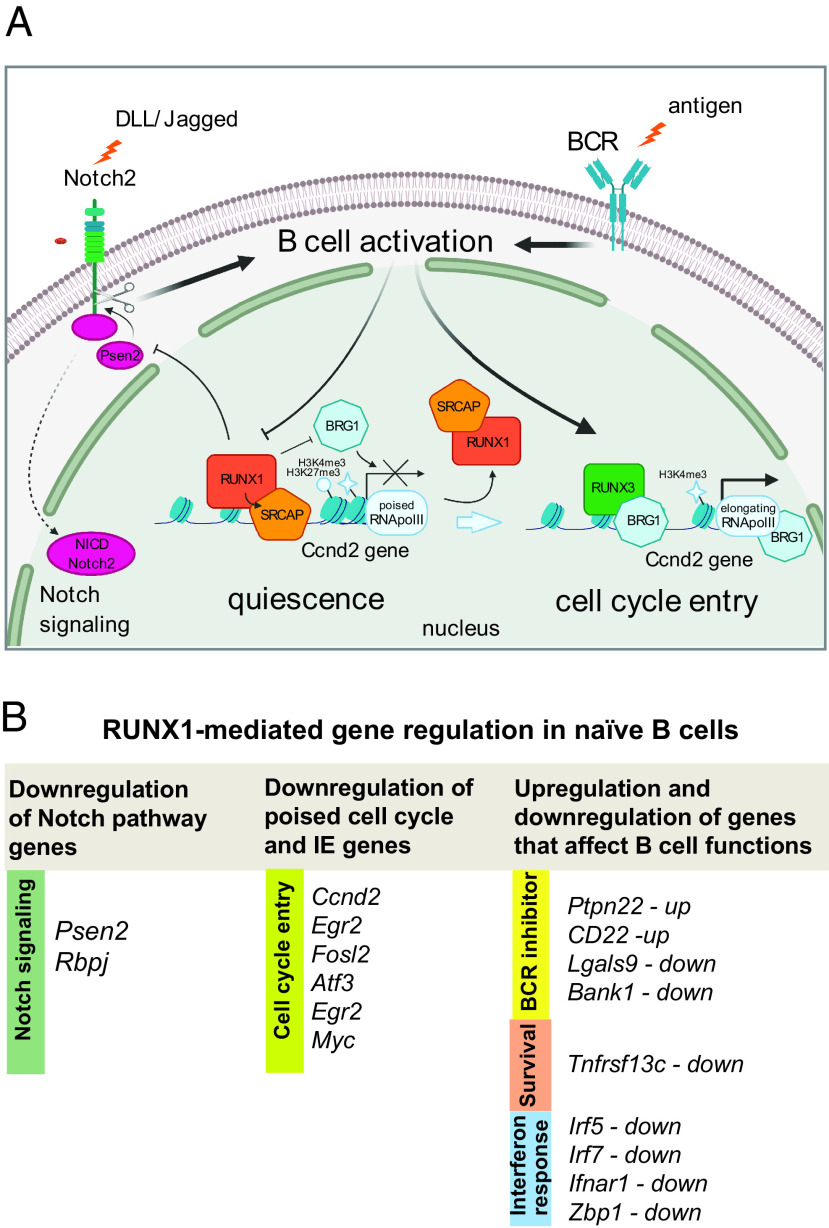
RUNX1-mediated regulation of gene expression affecting activation of naive B cells. (**A**) Schematic representation of the switch between RUNX1- and RUNX3-mediated gene regulation during activation of quiescent splenic B cells. The *Ccnd2* gene is shown as an example of a poised bivalent gene marked by histones H3K4me3 and H3K27me3, where RUNX1 recruits SRCAP and blocks binding of BRG1. This leads to reduced expression in resting B cells and immediately after BCR-mediated activation. After activation through stimulation of the BCR, RUNX1 is replaced at the promoter by RUNX3, which enhances binding of BRG1 and leads to full activation of *Ccnd2* expression. The diagram also shows the inhibitory effect of RUNX1 on Notch signaling through downregulation of *Psen2* expression. (**B**) Summary of the main genes affecting B cell activation and survival that are regulated by RUNX1 in naive B cells.

In addition to genes that affect cell survival and the BCR, the *Runx1* knockout also upregulates expression of a number of genes that are involved in the IFN response (Table I, Fig. 7A). These include the genes encoding the IFN regulatory factors IRF5 and IRF7, the IFN-α receptor gene *Ifnar1*, the *Zbp1* gene, which encodes DNA-dependent activator of IFN-regulatory factors, and *Stat1*, which encodes a signaling factor that enhances IFN-induced gene expression. Type I IFNs promote B cell activation (reviewed in Ref. 68), and our results imply that RUNX1 acts to dampen down this response, further supporting the conclusion that RUNX1 regulates multiple pathways to modulate stimulation of the B cell response (Fig. 8).

## Discussion

RUNX1 is known to be a critical regulator of gene expression during embryonic and adult hematopoiesis. The results described in this article extend our knowledge of the functions of RUNX1 by showing that it regulates a transcriptional program in naive resting B cells that reduces responsiveness of the cells to BCR and CD40 stimulation and delays entry into S-phase in response to these stimuli. Our results also provide evidence that the RUNX1 transcriptional program enhances proliferation of naive B cells in response to LPS stimulation, suggesting that it is involved in mediating the balance between BCR- and TLR-mediated responses of naive B cells.

Cell cycle and immediate early genes are a major category of genes that were affected by the RUNX1 knockout. Analysis of expression profiles revealed a rapid increase in transcript levels and enhancement of the rate at which transcription of cell cycle and immediate early genes increases in response to BCR stimulation in the knockout cells. The conclusion that this indicates a role for RUNX1 in regulating poising of these genes is reinforced by the presence of high levels of RNA Pol II at their promoters in wild-type resting B cells and relatively low levels in the body of the genes. Further support for poising of the cell cycle and immediate early genes comes from the observation that the promoters are marked with bivalent H3K4me3 and H3K27me3 histone modifications, which have been found to be associated with promoter poising in stem cells and in differentiated T cells and neuronal cells (40, 42, and reviewed in Ref. 46). Cyclin D2 has been shown to play an important role in B cell activation (30, 31, 69), and ectopic overexpression of *Ccnd2* has been shown to induce postmitotic cardiomyocytes to enter the cell cycle (70). Our data lead us to conclude that RUNX1-mediated downregulation of cyclin D2 expression plays an important role in controlling the rate of entry of naive B cells into S-phase.

Binding of RUNX1 to the poised cell cycle and immediate early genes occurs in conjunction with the chromatin remodeler SRCAP with particularly strong binding observed at distally located enhancer/silencer elements. BCR-mediated activation leads to a switch between binding of RUNX1 and its paralogue RUNX3 and between SRCAP and the SWI/SNF remodeling component BRG1. The hypothesis that RUNX1 recruits SRCAP to enhancers and promoters is supported by the strong colocalization of SRCAP binding with RUNX1, the reduction in binding of SRCAP at the *Ccnd2* and *Rbpj* promoters in the *Runx1* c-k/o resting B cells, and the fact that RUNX1 and SRCAP show a coordinated reduction in binding during B cell activation. It is noteworthy that RUNX3 has been reported to recruit BRG1 to gene promoters (56), suggesting that the switch from RUNX1 to RUNX3 binding plays a direct causal role in the SRCAP to BRG1 switch. However, the finding that BRG1 binding is increased in the knockout not only on BCR activation but also at the resting B cell stage, when RUNX3 protein is not yet expressed, indicates that BRG1 binding is downregulated by RUNX1 independently of RUNX3. These results also show that the increase in BRG1 binding that is observed at poised promoters in the knockout resting B cells persists after BCR-mediated activation by RUNX1. BRG1 has been shown to assist RNA Pol II in traversing a nucleosomal barrier, thereby promoting transcriptional elongation (71). Our findings support the idea that BRG1 and RUNX1 have opposing effects on poised promoters in resting B cells, with RUNX1 downregulating binding of BRG1, thereby reducing the rate of transition from a poised to an elongating transcriptional state (illustrated schematically in (Fig. 8A).

Interestingly, BRG1 has been shown previously to act in opposition to the repressive effects of the Autoimmune Regulator protein to poise peripheral-tissue-specific genes for expression during differentiation of medullary thymic epithelial cells (43). Low-level expression of peripheral-tissue-specific genes in medullary thymic epithelial cells allows their protein products to be presented on the cell surface, where they induce tolerance. The balance between Autoimmune Regulator and BRG1 binding is critical for achieving the correct timing and level of expression required to achieve this. BRG1 has also been implicated in gene poising in mouse ES cells as part of the ES cell BRG1/Brahma-associated factors (esBAF) remodeling complexes (72), and our data suggest that it has a similar role in resting B cells.

In contrast, the knockout of RUNX1 does not significantly affect enrichment of the PRC2-dependent modification, H3K27me3, at the *Ccnd2* gene, although levels of H3K27me3 were strongly decreased at both genes after activation. Levels of the PRC1 protein RING1B were increased on the *Ccnd2* gene in anti-IgM/IL-4–treated wild-type cells and in the *Runx1* c-k/o resting B cells. These results indicate that the PRC1 and PRC2 complexes are recruited independently of RUNX1 to the poised genes in B cells by as yet undetermined mechanisms. Binding of PRC1 independently of PRC2 has been shown to facilitate chromatin looping in mouse neural progenitor stem cells (50), so it is possible that increased binding of PRC1 at the *Ccnd2* gene during B cell activation acts to promote looping and stabilization of interactions between chromatin regulatory regions in the vicinity of the gene.

Our results also indicate that components of the NOTCH signaling pathway are affected by RUNX1. Expression of the γ-secretase catalytic subunit, *Psen2*, was strongly upregulated by the *Runx1* knockout in resting B cells. This was reflected in the increase in activated nuclear Notch2 protein that was observed in *Runx1* c-k/o resting B cells. Expression of the Notch effector, RBPJ, which binds to gene promoters in conjunction with activated Notch, was upregulated during activation of *Runx1* knockout B cells. The involvement of the Notch pathway in the modulating effect of RUNX1 on cell cycle entry was confirmed by the observation that premature entry of the *Runx1* knockout B cells into S-phase was reduced by treatment with a Notch inhibitor.

In addition to its effects on cell cycle and Notch pathway genes, our results identify genes that are either upregulated or downregulated by RUNX1, which are involved in a variety of B cell functions, including cell survival, BCR activation, and the type I IFN response. These genes do not, in general, show evidence of poising, and the major effect of RUNX1 binding is to increase or decrease the levels at which they are transcribed, before and immediately after activation. Overall, the known roles of the genes affecting B cell functions suggest that RUNX1 acts to reduce survival of FO B cells by downregulating levels of the BAFF receptor, as well as exerting a balancing effect on BCR signaling through upregulation of some genes that inhibit BCR signaling (*Ptpn22* and *Cd22*) and downregulation of others that also encode BCR inhibitors (*Trib1* and *Bank1*). In addition to these effects, our results indicate that RUNX1 has a generally inhibitory effect on expression of IFN response genes in resting B cells.

The gene regulation effects of RUNX1 that have been identified in this study are summarized in (Fig. 8B. Overall, they provide evidence that a primary function of RUNX1 is to act as a negative regulator of BCR-mediated B cell activation by altering the expression profile of poised genes that affect the dynamics of cell cycle entry and by reducing expression of Notch signaling pathway components. In addition to these effects, RUNX1 antagonizes the prosurvival effects of BAFF and exerts enhancing and inhibitory effects on the expression of genes that are known to reduce BCR responsiveness during the early stages of B cell activation in response to Ag. The effects of RUNX1 on the expression of multiple cell cycle and signaling pathway genes manifest themselves in the strong phenotype of accelerated cell cycle entry that is exhibited by *Runx1* knockout resting B cells in response to BCR stimulation. The number of RUNX1-regulated genes and their diverse functional roles in cell cycle, BCR, and Notch pathway regulation in B cells make it difficult to quantify the respective contributions of individual genes to the cell cycle phenotype. It is likely that, similar to many biological phenomena, the overall phenotype is the sum of effects from a number of different genes. Further studies will be required to dissect these different contributions.

Several of the genes that we have identified as targets for RUNX1 regulation in B cells have been linked to autoimmune diseases using genome-wide association study analysis (73), functional studies in animal models (74–77), or direct analysis in patients (68, 78). In addition to establishing a novel regulatory function for RUNX1 in B cell activation, our results suggest that changes to the expression and functioning of RUNX1 in B cells could have causal roles in human autoimmune disease.

## Supplementary Material

Data Supplement
