# Supplementary material for: RUNX1 Regulates a Transcription Program That Affects the Dynamics of Cell Cycle Entry of Naive Resting B Cells
Source: J Immunol. 2021 Dec 15;207(12):2976–91. doi: 10.4049/jimmunol.2001367 (PMC8675107; doi:10.4049/jimmunol.2001367)
Supplement: Data Supplement [file JI_2001367.zip › JI_2001367_Supplemental_1.pdf]

Thomsen et al

Supplemental Figures

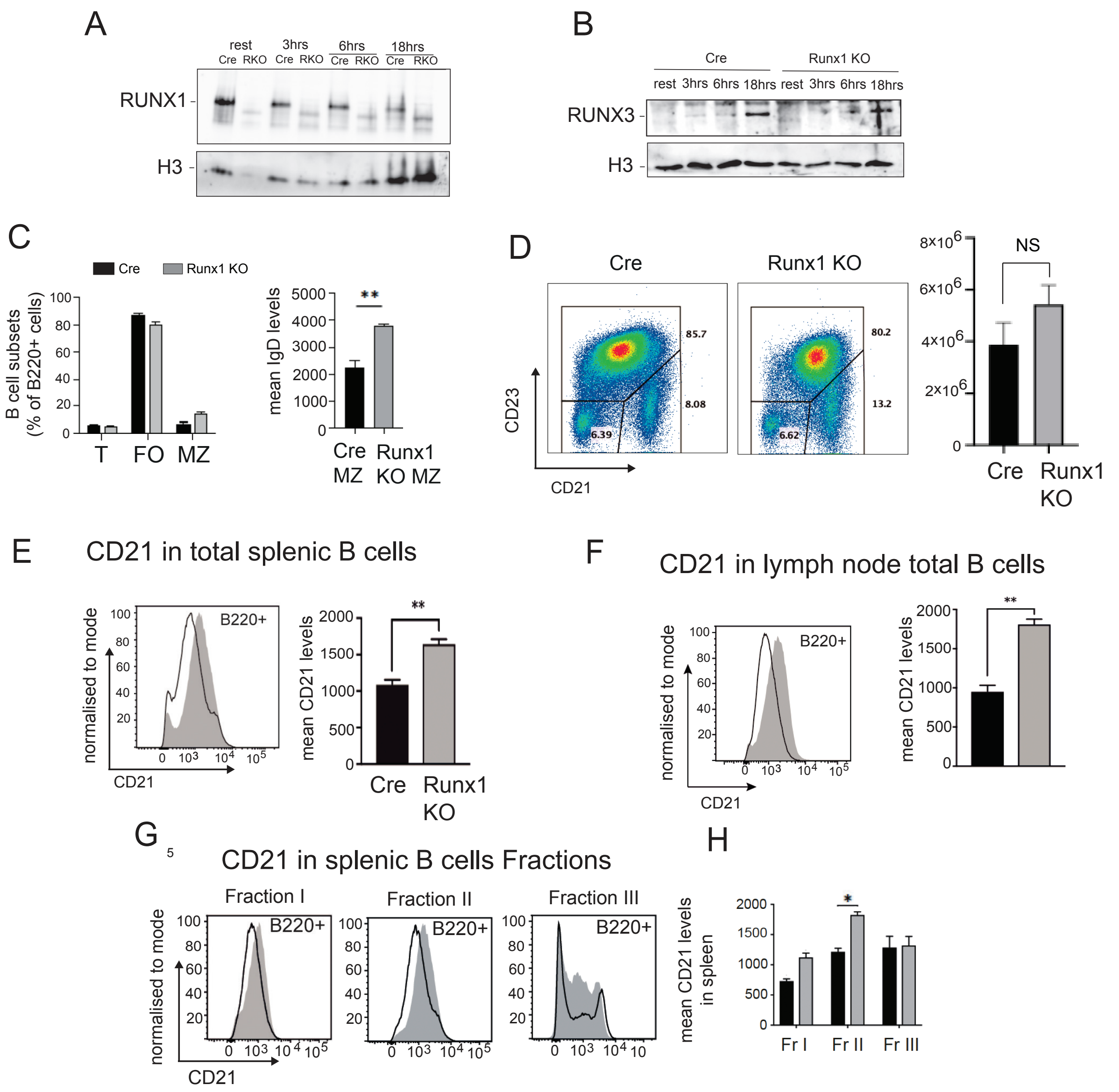

Supplementary Figure 1. **A, B**. Western Blot analysis of the levels of RUNX1 (A) and RUNX3 (B) in *Runx1* c-k/o (RKO) and CD23-cre (Cre) control resting B cells after activation with anti-IgM for 0-18hrs. **C**. Left panel; percentages of Transitional (T – CD23low/CD21low), Follicular (FO – CD23high/CD21mid-high) and Marginal Zone (MZ – CD23low/CD21high) B cells in splenic $B220^{+}$ cell populations. Right panel: Mean of IgD levels on the surface of MZ cells. Statistical calculations for panels C, D, E, F: error bars represent SEM. * $p \leq 0.05$, ** $p \leq 0.01$, NS: non-significant, Student's t test. n=3. **D**. Quantification of marginal zone (MZ) ($CD21^{hi}/CD23^{lo}$) cells in Cre-only and *Runx1* c-k/o spleens. The histogram shows the number of $CD21^{hi}/CD23^{lo}$ B cells for each genotype. **E**: Runx1 c-k/o splenic B cells express higher levels of surface CD21. Left panel: representative histogram of CD21 surface levels on B220+ splenic B cells. Y-axis represents frequency of the CD21 level on the X-axis shown as a percentage of maximum count. Right panel: mean CD21 surface levels on splenic B220+ cells. **F**. Runx1 c-k/o lymph-node B cells express higher levels of surface CD21.Left panel: Representative histogram: of CD21 level as a percentage of maximum count. Right panel: mean CD21 surface levels on B220+ lymph node B cells. **G.** Expression of CD21 in splenic B cell fractions: Representative histograms of CD21 surface levels in Fraction I-III cells from B220+ splenic B cells are shown. **H.** Mean CD21 surface levels on Fraction I-III spenic B cells from the analysis shown in (G). Values = mean ± SEM. Significance was calculated using one-way ANOVA followed by Tukey's multiple comparisons test. *p≤0.05.

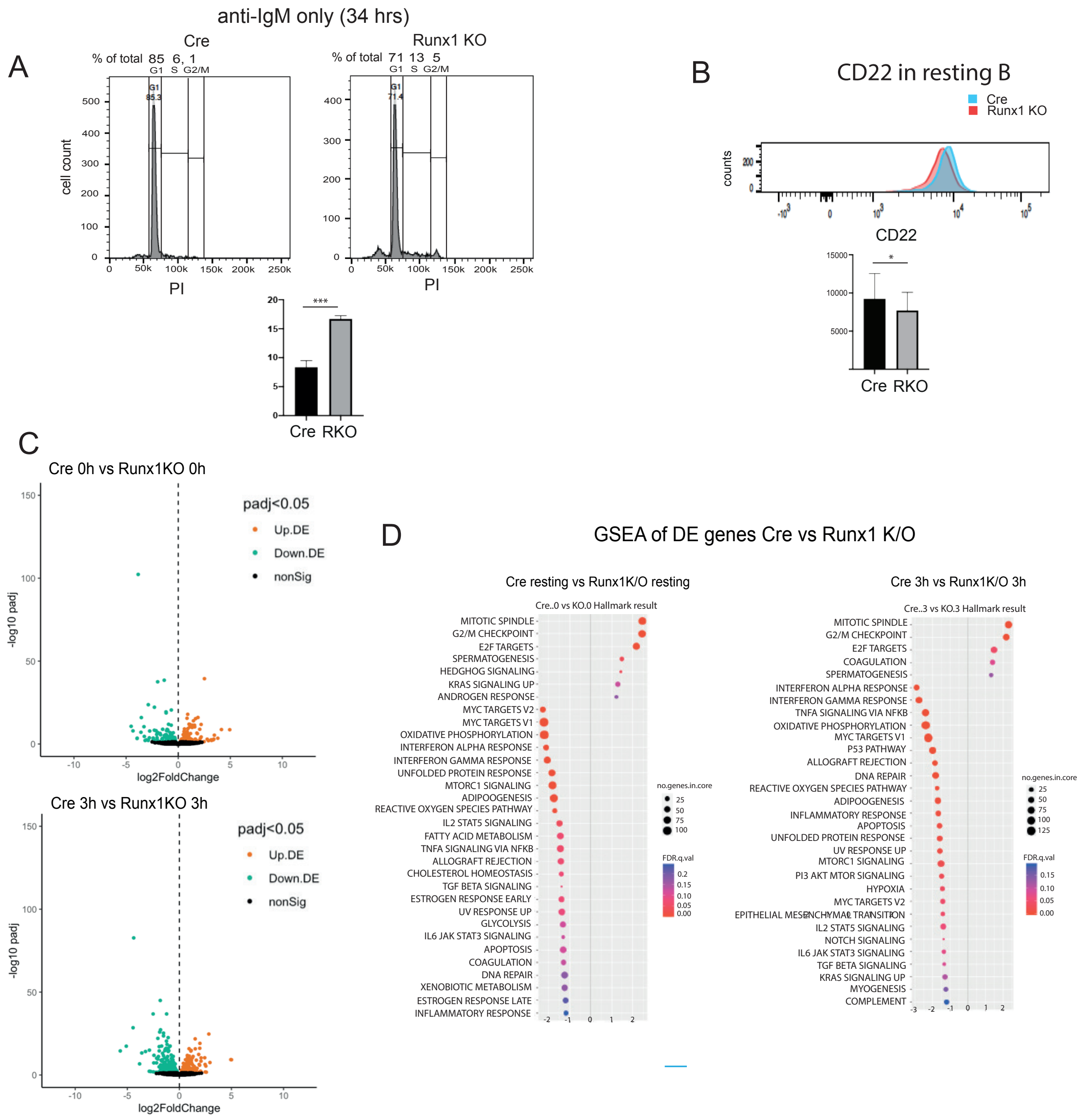

Supplementary Figure 2. A. Effect of the *Runx1* knockout on S-phase entry of resting B cells following stimulation of control CD23-cre (Cre) and *Runx1* c-k/o resting B cells with anti-IgM only. Proportions of cells in G1/S/G2-M were measured by PI staining as in Figure 1D. Top pane: Representative FACS analysis. Bottom panel: numbers of cell in G2/M. For (A), (B) and (B)Values = mean ± SEM. *** p≤0.001, Student's t-test, n= 3 **B.** FACS analysis of CD22 protein expression in Cre and Runx1 c-k/o resting B cells. Plots show expression in 3 biological replicates. **C**. 'Volcano plots' of statistical significance (adjusted p value) against fold- change of differentially expressed (DE) genes in Cre vs Runx1 ck/o B cell at the resting state (left panels) or after 3 hrs of activation with anti-IgM. **D**. Scatter plots illustrate enriched Runx1 c-k/o database pathways in resting B cells (left panel) and in 3hrs with anti-IgM activated cells (right panel). The vertical axis represents the enriched pathway categories and the horizontal axis represents the NES = Normalised Enrichment Score of the enriched pathways. The size and colour of dots represent the gene number and the range of p-values, respectively.

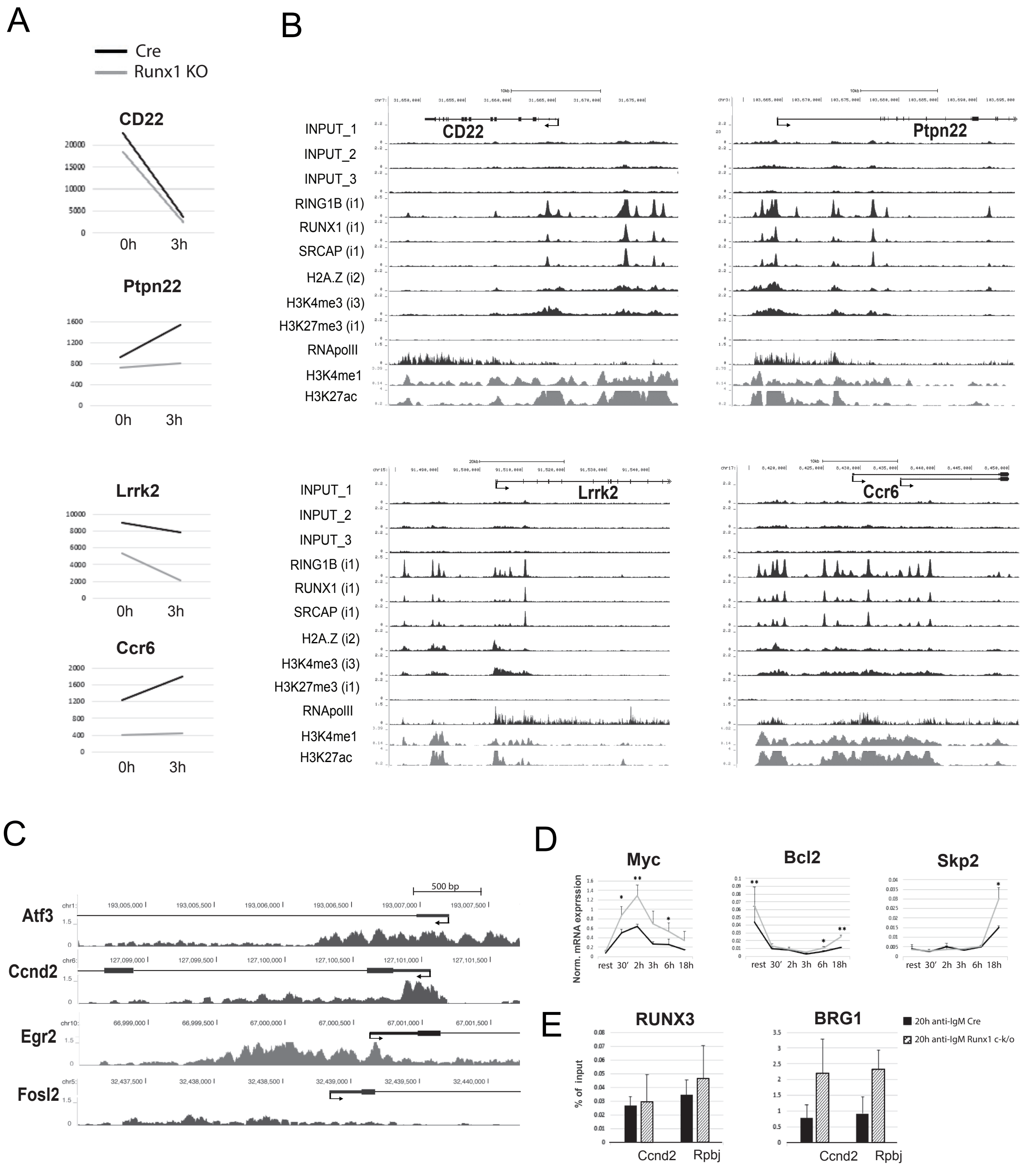

Supplementary Figure 3: **A**. BaseMean values for expression of genes with functional roles in B cells that are downregulated in the Runx1 c-k/o B cells (see Table I). Values were obtained from the RNAseq analysis at 0 and 3hrs anti-IgM + Il-4 treatment (see Table S1A and S1B). All values are the mean of 3 biological replicates. Adjusted p values for the differences in gene expression levels were < 0.05 for at least one of the points. **B**. ChIPseq tracks showing the profiles of factor binding and histone modification in resting B cells of the genes shown in (A). **C.** Close up of RNA Pol II occupancy levels at poised genes that are regulated by RUNX1 (the full set of ChIP-seq tracks for these genes are shown in Figure 3). **D**. RT-qPCR analysis of expression of the Notch target genes Myc, Bcl2 and Skp2 in resting B cells and following stimulation with anti-IgM for the times shown on the x-axes. The y-axes show normalised RNA expression. Values are mean ± SD. * p≤0.5, ** p≤0.01. Student's t-test, n = 3. **E.** qPCR-ChIP analysis of binding of RUNX3 and BRG1 to to the *Ccnd2* and *Rbpj* promoters in Cre-only (Cre) and *Runx1* c-k/o B cells after 20 hrs stimulation with anti-IgM + Il-4.
